# Detecting and correcting the binding-affinity bias in ChIP-seq data using inter-species information

**DOI:** 10.1186/s12864-016-2682-6

**Published:** 2016-05-10

**Authors:** Martin Nettling, Hendrik Treutler, Jesus Cerquides, Ivo Grosse

**Affiliations:** Institute of Computer Science, Martin Luther University, Halle (Saale), Germany; Leibniz Institute of Plant Biochemistry, Halle (Saale), Germany; IIIA-CSIC, Campus UAB, Barcelona, Spain; German Centre for Integrative Biodiversity Research (iDiv) Halle-Jena-Leipzig, Leipzig, Germany

**Keywords:** Binding-affinity bias, ChIP-seq, Phylogenetic footprinting, Evolution, Transcription factor binding sites, Gene regulation

## Abstract

**Background:**

Transcriptional gene regulation is a fundamental process in nature, and the experimental and computational investigation of DNA binding motifs and their binding sites is a prerequisite for elucidating this process. ChIP-seq has become the major technology to uncover genomic regions containing those binding sites, but motifs predicted by traditional computational approaches using these data are distorted by a ubiquitous binding-affinity bias. Here, we present an approach for detecting and correcting this bias using inter-species information.

**Results:**

We find that the binding-affinity bias caused by the ChIP-seq experiment in the reference species is stronger than the indirect binding-affinity bias in orthologous regions from phylogenetically related species. We use this difference to develop a phylogenetic footprinting model that is capable of detecting and correcting the binding-affinity bias. We find that this model improves motif prediction and that the corrected motifs are typically softer than those predicted by traditional approaches.

**Conclusions:**

These findings indicate that motifs published in databases and in the literature are artificially sharpened compared to the native motifs. These findings also indicate that our current understanding of transcriptional gene regulation might be blurred, but that it is possible to advance this understanding by taking into account inter-species information available today and even more in the future.

**Electronic supplementary material:**

The online version of this article (doi:10.1186/s12864-016-2682-6) contains supplementary material, which is available to authorized users.

## Background

Predicting transcription factor binding sites and their motifs is essential for understanding transcriptional gene regulation and thus of importance in almost all areas of modern biology, medicine, and biodiversity research [1, 2]. Countless approaches exist for predicting motifs from these genomic regions [3–6], but predicting motifs from ChIP-seq data and similar experimental data is hampered by the contamination with false positive genomic regions as well as the enrichment of high-affinity binding sites [7–9].

The contamination with false positive genomic regions is caused by at least three reasons. First, the transcription factor or other DNA binding protein pulled down by immunoprecipitation may not bind directly to the binding site [10]. Second, ChIP-seq target regions may not contain a binding site due to experimental settings such as sequencing depth or DNA fragment length [11, 12]. Third, false positive regions may be predicted in the subsequent ChIP-seq data analysis due to never perfect analysis pipelines and too low signal cutoff thresholds [8]. These three effects may lead to the selection of false positive ChIP-seq regions that do not contain at least one binding site.

The enrichment of high-affinity binding sites is caused by at least two reasons. First, most antibodies have a preference of binding high-affinity binding sites with a higher probability than low-affinity binding sites, causing the set of binding sites bound in the ChIP-seq experiment to be partially different from the set of binding sites bound in vivo [13, 14]. Second, true positive regions with low-affinity binding sites are rejected due to too high signal cutoff thresholds [5, 8]. These two effects may lead to an under-representation of low-affinity binding sites and an over-representation of high-affinity binding sites in ChIP-seq regions.

Taken together, the contamination with false positive genomic regions leads to the *contamination bias* [15] and thus to the prediction of artificially softened motifs, whereas the enrichment of sequences with high-affinity binding sites leads to the *binding-affinity bias* [16] and thus to the prediction of artificially sharpened motifs. Neglecting these effects leads to distorted motifs and could potentially affect all downstream analyses [17–20]. Existing approaches for predicting motifs are capable of detecting and correcting the contamination bias, which has been found to increase the quality of motif prediction considerably [8, 21, 22], and here we investigate the possibility of detecting and correcting the binding-affinity bias.

Detecting the binding-affinity bias seems impossible based on sequence data from one species alone, but it seems possible based on inter-species information. This is possible due to the fact that the binding-affinity bias is stronger in the target regions of the ChIP-seq experiment in the reference species than in orthologous regions of phylogenetically related species. This stronger binding-affinity bias yields more biased motifs in the reference species than in phylogenetically related species, and this difference may be used for detecting and potentially correcting the binding-affinity bias.

Phylogenetic footprinting models typically (i) take into account ChIP-seq data of only one species and (ii) do not take into account heterogeneous substitution rates among different DNA regions, heterotachious evolution of DNA regions, and loss-of-function mutations in binding sites. The consideration of (i) ChIP-seq data of more than one species and (ii) heterogeneity, heterotachy, and loss-of-function mutations are likely to improve both phylogenetic footprinting as well as the detection and correction of the binding-affinity bias, but in this work we investigate if the detection and correction of this bias is possible based on (i) ChIP-seq data of only one species and (ii) a simple phylogenetic footprinting model that neglects heterogeneity, heterotachy, and loss-of-function mutations.

We first investigate if the effect of observing more biased motifs in the reference species than in phylogenetically related species is measurable beyond statistical noise in target regions of five ChIP-seq data sets of human and in orthologous regions of monkey, dog, cow, and horse. We then develop a phylogenetic footprinting model that incorporates the binding-affinity bias, investigate if this model improves or deteriorates motif prediction compared to traditional models that do not incorporate it, and compare the motifs predicted with and without the correction of the binding-affinity bias.

## Results and discussion

In subsection “Using sequence-information of phylogenetically related species to detect the binding-affinity bias”, we describe the basic idea of how the binding-affinity bias could be detected based on inter-species information using a toy example. In the remaining subsections we perform three studies based on ChIP-seq data sets of five transcription factors and on multiple alignments of the human ChIP-seq target regions with orthologous regions from monkey, dog, cow, and horse. In subsection “Decrease of information contents in motifs from phylogenetically related species” we investigate if the effect of observing more biased motifs in the reference species than in phylogenetically related species is measurable in these five data sets. In subsection “Modeling the binding-affinity bias increases classification performance”, we investigate if a correction of the binding-affinity bias leads to an improvement or a deterioration of the classification performance. In subsection “Modeling the binding-affinity bias leads to softened motifs”, we compare the sequence motifs predicted with and without the correction of the binding-affinity bias.

### Using sequence-information of phylogenetically related species to detect the binding-affinity bias

**Fig. 1:**
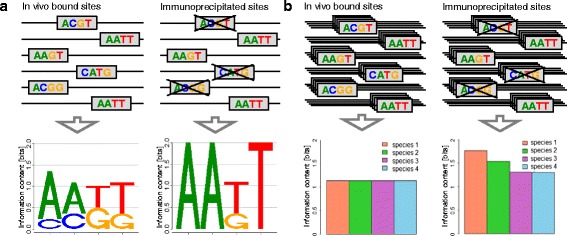
Influence of binding-affinity bias on information content. **a** Binding-affinity bias in the reference species. The left column shows binding sites bound in vivo as well as the sequence logo. In the right column, enrichment of high-affinity binding sites by chromatin immunoprecipitation leads to a different motif with higher information content. **b** Binding-affinity bias in the reference species and three phylogenetically related species. The left column shows binding sites bound in vivo and the information content of the species–specific motifs. In the right column, the enrichment of high-affinity binding sites in the reference species and the other three species leads to different motifs with different information content in each species. The effect of this enrichment decreases with the phylogenetic distance from the reference species as reflected by decreasing information contents. Please find the sequences of all species in Table 1

Detecting and correcting the binding-affinity bias might be possible because the binding-affinity bias inherent to the ChIP-seq experiment in the reference species (Fig. 1a) is stronger than the indirect binding-affinity bias in orthologous regions from phylogenetically related species. Under this assumption, the information content of the predicted motifs [23] should decrease with the phylogenetic distance from the reference species due to the increasing number of mutations.

**Table 1:**
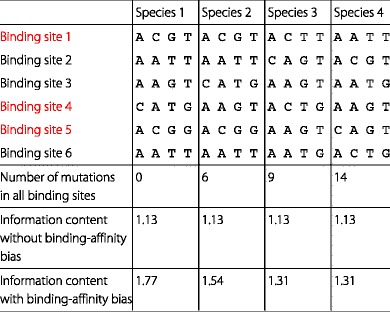
Influence of binding-affinity bias on information content. We illustrate the effect of binding-affinity bias with the given toy example of a ChIP-seq experiment for six binding sites in four species. Due to low binding-affinity, red binding sites are insufficiently bound. This results in the absence of red binding sites in the measured data which we denote binding-affinity bias. Binding sites with low binding-affinity typically comprise dissimilar bases in contrast to black binding sites with high affinity and common bases. The absence of red binding sites leads to a sharpening of the resulting motif, which we indicate using the information content. The information content without binding-affinity bias is equal in all species, whereas the information content with binding-affinity bias increases in all species. The vital point is that the effect of binding-affinity bias decreases with phylogenetic distance, which involves an increasing number of mutations. Please find a visualization of this toy example in Fig. 1 b

To illustrate this idea, we present a toy example consisting of six binding sites from four phylogenetically related species in Fig. 1b and Table 1. In this toy example, we assume an exaggerated binding-affinity bias of three high-affinity binding sites captured by the ChIP-seq experiment and three low-affinity binding sites not captured by the ChIP-seq experiment. In real world applications the native motif is unknown and the motif predicted on the available data is biased to an unknown degree. In the presented toy example, however, the native motif is considered to be known so that the effect of the binding-affinity bias on the motifs of the reference species (species 1) and the phylogenetically related species (species 2, 3, and 4) can be illustrated.

The motif predicted from the three target regions containing high-affinity binding sites is strongly biased in reference species 1, and it is impossible to predict the native motif from only those three target regions. However, a shadow of this strong binding-affinity bias also exists in orthologous regions of species 2, 3, and 4, so the motifs predicted from these orthologous regions in species 2, 3, and 4 are biased, too. This bias in species 2, 3, and 4, however, is weaker than the bias in reference species 1, and this difference can be exploited for detecting and correcting the binding-affinity bias and for predicting the native motif from the three target regions of high-affinity binding sites in reference species 1 and their orthologous regions in species 2, 3, and 4.

Specifically, the binding-affinity bias introduced by the ChIP-seq experiment in reference species 1 causes a strong increase of the information content of the predicted motif (1.77 bit) compared to the native motif (1.13 bit). The shadow of the binding-affinity bias in species 2, 3, and 4 also causes an increase of the information contents of the motifs predicted in species 2 (1.54 bit), species 3 (1.31 bit), and species 4 (1.31 bit), but this increase in species 2, 3, and 4 is smaller than in reference species 1 (Table 1 and Fig. 1b). The increase of information content decreases with the number of observed mutations and thus the phylogenetic distance of species 2, 3, and 4 to reference species 1 in which the ChIP-seq experiment has been performed. Hence, the observation of a decreased information content of motifs predicted in orthologous regions of phylogenetically related species compared to the information content of the motif predicted in the reference species could indicate the presence of a binding-affinity bias and possibly allow the correction of that bias.

### Decrease of information contents in motifs from phylogenetically related species

**Fig. 2:**
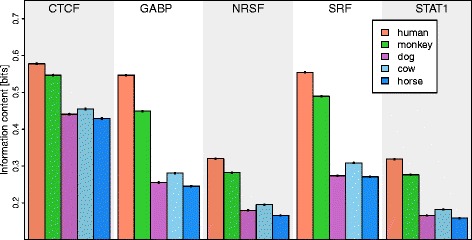
Mean information content and standard error for motifs of five transcription factors in five species. The information content of motifs in the reference species (human) is significantly higher compared to the four phylogenetically related species (*p*<1.8×10^−14^). The information content typically decreases with the phylogenetic distance from the reference species

We investigate this hypothesis on human ChIP-seq data of five transcription factors [10, 24] and multiple alignments of the human ChIP-seq target regions with orthologous regions from monkey, dog, cow, and horse [25] (“Data” Methods). We calculate the information contents of motifs from human (reference species), monkey, dog, cow, and horse for each of the five data sets (“Decrease of information contents in motifs from related species” Methods) and present the results in Fig. 2. We find for each of the five data sets that the information content of the motif from the reference species is significantly higher (*p*<1.83×10^−14^, Wilcoxon Signed-Rank Test, Additional file 1: Table S1) compared to the information contents of the motifs from monkey, dog, cow, and horse.

### Modeling the binding-affinity bias increases classification performance

Motivated by this observation, we develop a phylogenetic footprinting model capable of taking into account the contamination bias (${\mathcal {M}_{-}^{\mathrm {C}}}$), the binding-affinity bias (${\mathcal {M}_{\text {BA}}^{-}}$), neither one or the other ${\mathcal {M}^{-}_{-}}$, or both (${\mathcal {M}_{\text {BA}}^{\mathrm {C}}}$) (“Modeling the binding-affinity bias” Methods and Additional file 1: Section 1). In order to study to which degree these models are capable of modeling multiple alignments originating from ChIP-seq data, we consider the principle of parsimony [26], which states that the simplest of competing explanations is the most likely to be correct. As the new model ${\mathcal {M}_{\text {BA}}^{\mathrm {C}}}$ is more complex than the traditional model ${\mathcal {M}_{-}^{\mathrm {C}}}$, we should accept it only if it provides a more accurate representation of the data. A standard approach for measuring how accurately a model represents a data set is to measure its performance of classifying, in this case, motif-bearing and non-motif-bearing alignments, and a standard approach for measuring classification performance is stratified repeated random sub-sampling validation (“Measuring classification performance” Methods, Fig. 5).

**Fig. 3:**
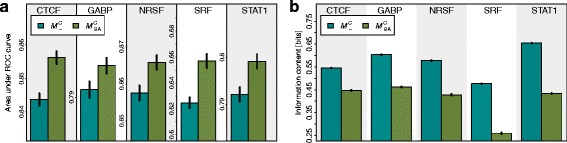
Comparison of models ${\mathcal {M}_{-}^{\mathrm {C}}}$ and ${\mathcal {M}_{\text {BA}}^{\mathrm {C}}}$. **a** Mean classification performance and standard error of the two models ${\mathcal {M}_{-}^{\mathrm {C}}}$ and ${\mathcal {M}_{\text {BA}}^{\mathrm {C}}}$ quantified by the area under the receiver operating characteristic curve. We find for each of the five data sets a significantly increased classification performance for ${\mathcal {M}_{\text {BA}}^{\mathrm {C}}}$ compared to ${\mathcal {M}_{-}^{\mathrm {C}}}$. Examples for ROC curves are shown in Additional file 1: Figure S10, Figure S11, Figure S12, Figure S13, Figure S14 and Figure S15. **b** Mean information content and standard error of the motifs predicted by the two models ${\mathcal {M}_{-}^{\mathrm {C}}}$ and ${\mathcal {M}_{\text {BA}}^{\mathrm {C}}}$. We find for each of the five data sets a significantly decreased information content in motifs predicted by ${\mathcal {M}_{\text {BA}}^{\mathrm {C}}}$ compared to ${\mathcal {M}_{-}^{\mathrm {C}}}$ (*p*<4.0×10^−18^)

Using this approach we measure the performance of the four models ${\mathcal {M}^{-}_{-}}$, ${\mathcal {M}_{\text {BA}}^{-}}$, ${\mathcal {M}_{-}^{\mathrm {C}}}$, and ${\mathcal {M}_{\text {BA}}^{\mathrm {C}}}$ to classify each of the five data sets against the other four. Fig. 3a shows that ${\mathcal {M}_{\text {BA}}^{\mathrm {C}}}$ yields a higher classification performance than ${\mathcal {M}_{-}^{\mathrm {C}}}$ in all five data sets (*p*<2.3×10^−17^, Wilcoxon Signed-Rank Test, Additional file 1: Table S2), indicating that the new model ${\mathcal {M}_{\text {BA}}^{\mathrm {C}}}$ is more realistic than the traditional model ${\mathcal {M}_{-}^{\mathrm {C}}}$. We also find that ${\mathcal {M}_{\text {BA}}^{-}}$ yields a significantly higher classification performance than ${\mathcal {M}_{-}^{\mathrm {C}}}$ in all five data sets (*p*<1.8×10^−17^, Wilcoxon Signed-Rank Test), which indicates that taking into account the binding-affinity bias has a larger impact on the classification performance than taking into account the contamination bias (Additional file 1: Figure S1, Figure S2, Figure S10, Figure S11, Figure S12, Figure S13, Figure S14, Figure S15 and Figure S16).

### Modeling the binding-affinity bias leads to softened motifs

Next, we investigate the information contents of the corrected motifs predicted by models ${\mathcal {M}_{\text {BA}}^{-}}$ and ${\mathcal {M}_{\text {BA}}^{\mathrm {C}}}$ that take into account the binding-affinity bias and the traditional motifs predicted by models ${\mathcal {M}^{-}_{-}}$ and ${\mathcal {M}_{-}^{\mathrm {C}}}$ that neglect this bias. Fig. 3b shows that the information contents of motifs predicted by ${\mathcal {M}_{-}^{\mathrm {C}}}$ are significantly higher than the information contents of motifs predicted by ${\mathcal {M}_{\text {BA}}^{\mathrm {C}}}$ (*p*<4.0×10^−18^, Wilcoxon Signed-Rank Test). We also find that the information contents of motifs predicted by ${\mathcal {M}^{-}_{-}}$ are higher than the information contents of motifs predicted by ${\mathcal {M}_{\text {BA}}^{\mathrm {C}}}$ (*p*<4.0×10^−18^, Wilcoxon Signed-Rank Test, Additional file 1: Table S4), stating that the binding-affinity bias is stronger than the contamination bias. Equivalently, this states that the joint effect of both biases leads to an artificial sharpening of the motifs and an artificial overestimation of the binding affinities (Additional file 1: Figure S3, Figure S4, Figure S17, Figure S18).

**Fig. 4:**
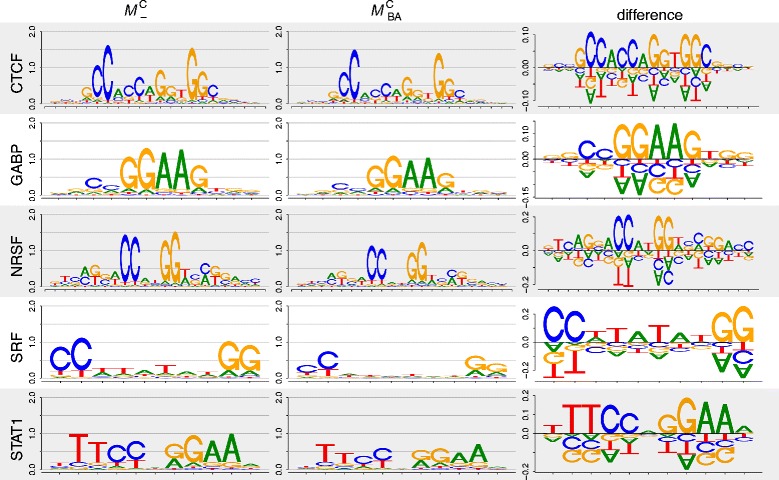
Differences of motifs predicted by ${\mathcal {M}_{-}^{\mathrm {C}}}$ and ${\mathcal {M}_{\text {BA}}^{\mathrm {C}}}$. The height of the base stacks is quantified by the Jensen-Shannon divergence, where high base stacks represent high motif differences. We find significant motif differences exceeding 0.1 bit for all five data sets (Additional file 1: Figure S5, Figure S6, Figure S7, Figure S8 and Figure S9)

Finally, we inspect the differences of the corrected motifs predicted by ${\mathcal {M}_{\text {BA}}^{-}}$ and ${\mathcal {M}_{\text {BA}}^{\mathrm {C}}}$ and the traditional motifs predicted by ${\mathcal {M}^{-}_{-}}$ and ${\mathcal {M}_{-}^{\mathrm {C}}}$. Fig. 4 shows the differences between the base distributions of pairs of motifs for ${\mathcal {M}_{-}^{\mathrm {C}}}$ and ${\mathcal {M}_{\text {BA}}^{\mathrm {C}}}$ by difference logos (“Visualizing motif differences with DiffLogo” Methods). We find for each of the five data sets that the corrected motifs are softer than the traditional motifs distorted by the binding-affinity bias. Specifically, we find that the amount of decrease of the most abundant bases in the corrected motifs compared to the traditional motifs is roughly proportional to the base abundance, whereas the increase of the remaining bases is not proportional to the base abundance. Hence, the corrected motifs are not simply a uniformly softened version of the traditional motifs, but motifs with different degrees of dissimilarity at different positions (Additional file 1: Figure S5, Figure S6,Figure S7, Figure S8 and Figure S9).

## Conclusions

We studied the possibility of detecting and correcting the binding-affinity bias in ChIP-seq data using inter-species information. We found that the fact that this bias is stronger in target regions of the reference species than its shadow in orthologous regions of phylogenetically related species enables the detection and correction of this bias. We proposed a phylogenetic footprinting model capable of taking into account the binding-affinity bias in addition to the contamination bias, and we applied this model and its three special cases that neglect one of the two biases or both to five ChIP-seq data sets. We found by stratified repeated random sub-sampling validation that taking into account the binding-affinity bias always improves motif prediction, that the motif binding-affinity bias leads to a distortion of motifs that is even stronger than the distortion caused by the contamination bias, and that the corrected motifs are typically softer than those predicted by traditional approaches. The comparison of corrected and traditional motifs showed small but noteworthy differences, suggesting that the refinement of traditional motifs from databases and from the literature might lead to the prediction of novel binding sites, *cis*-regulatory modules, or gene-regulatory networks and might thus advance our attempt of understanding transcriptional gene regulation as a whole.

## Methods

In this section we describe “Decrease of information contents in motifs from related species” (i) the determination of the information contents of motifs in the reference species and phylogenetically related species, “Modeling the binding-affinity bias” (ii) the phylogenetic footprinting model that can take into account the binding-affinity bias, the contamination bias, neither one or the other, or both, “Measuring classification performance” (iii) the measurement of the classification performance of these four phylogenetic footprinting models using stratified repeated random sub-sampling validation, and “Visualizing motif differences with DiffLogo” (iv) the visualisation of differences between the corrected and the traditional motifs.

### Decrease of information contents in motifs from related species

We determine the information content *I*(*P*) of a motif *P* as described in [23]:

1 $$ \begin{aligned} H_{\ell}(P) &= \log_{2}(|\mathcal{A}|) - \sum_{a \in \mathcal{A}} p_{\ell,a} \cdot \log_{2}(p_{\ell,a}) \\ I(P) &= \sum_{\ell = 1}^{W} H_{\ell}(P), \end{aligned}  $$

where $\mathcal {A} = {A,C,G,T}$ is the alphabet, *p*_*ℓ*,*a*_ is the probability of base *a* at position *ℓ* in motif *P*, and *H*_*ℓ*_(*P*) denotes the information content of position *ℓ* in motif *P*.

We measure the information contents of motifs in five species using repeated random sub-sampling as follows. Initially, we choose one motif for each of the transcription factors CTCF, GABP, NRSF, SRF, and STAT1 from the JASPAR database, namely MA0139.1 for CTCF, MA0062.2 for GABP, MA0138.2 for NRSF, MA0083.2 for SRF, and MA0137.3 for STAT1 [27]. In the first step, we generate a test set from the set of positive alignments (Table 2) by removing randomly 200 alignments. In the second step, we predict for each transcription factor one binding site per target region in all target regions of the reference species (human) in the corresponding test data set, extract the predicted binding sites from the reference species as well as the binding sites at the same positions in the orthologous regions, and calculate for each species the information content of the resulting motif as specified above. We perform both steps 100 times and report the mean and standard error of the information content for each of the five species.

**Table 2:** Data set statistics for human ChIP-seq data. For each of the five transcription factors (TFs) CTCF, GABP, NRSF, SRF, and STAT1, we specify the (i) average length of transcription factor binding site (TFBS), the (ii) number of alignments, and the (iii) average length of alignments

TF	TFBS length	Number of alignments	Avg. length
CTCF	20 bp	467	213 bp
GABP	12 bp	451	236 bp
NRSF	21 bp	460	245 bp
SRF	12 bp	394	242 bp
STAT1	11 bp	360	244 bp

### Modeling the binding-affinity bias

In this section we describe the probabilistic model for modeling the binding-affinity bias as a data generating process. A derivation of the log-likelihood for motif-bearing and non-motif-bearing alignments can be found in Additional file 1: Section 1.

Let *O* be the number of species. A data set comprises *N* independent multiple sequence alignments. We use *X*_*n*_ to refer to the *n*-th sequence alignment. Every alignment is formed by *O* sequences. The *o*-th sequence is denoted by $X_{n}^{.,o}$. By convention, the reference species (that in which the selection process has taken place) is species 1. Each sequence of alignment *X*_*n*_ is composed of *L*_*n*_ nucleotides. We denote by $X_{n}^{u,o}$ the *u*-th nucleotide of the *o*-th sequence of the *n*-th alignment. All nucleotides are presented by the set $\mathcal {A} = \{A,C,G,T\}$.

We assume the existence of a common ancestor of all of *O* species. The sequence of the common ancestor of the *n*-th alignment is a hidden variable *Y*_*n*_, with ${Y_{n}^{u}}$ representing its *u*-th nucleotide. The substitution probability that nucleotide ${Y_{n}^{u}}$ is substituted by the nucleotide $X_{n}^{u,o}$ is denoted by the variable *γ*_*o*_.

An alignment *X*_*n*_ may contain a binding site or not. This is denoted by the variable *M*_*n*_. The length of the binding site is denoted by the variable *W* and the position of the binding site in alignment *X*_*n*_ is denoted by the variable *ℓ*_*n*_.

The *n*-th alignment *X*_*n*_ is sampled as follows. The first decision to be made is whether or not the alignment contains a binding site. This is denoted by variable *M*_*n*_ which follows a Bernoulli distribution with parameter 1−*α*. Thus, whenever variable *M*_*n*_ is equal to 1 (${M_{n}^{1}}$), the alignment contains a binding site and when *M*_*n*_ is equal to 0 (${M_{n}^{0}}$), it does not.

Thus, parameter *α* is the probability that alignment *X*_*n*_ contains no binding site. If *α* equals 0, the sampled data is uncontaminated, because all alignments contain a copy of the binding site. The larger the value of *α*, the higher the percentage of non motif-bearing alignments in the sampled data. A value of *α* equal to 1 models a data set where no binding sites are present.

Next we introduce the data generating process for non-motif-bearing alignments and later we explain that for motif-bearing alignments.

1. Sample the primordial sequence as follows: For each position *u* of the sequence sample nucleotide ${Y_{n}^{u}}$ from the background equilibrium distribution *π*_0_ independent of the previous nucleotides.
2. For each of the descent species *o*∈{1,…,*O*}, sample its sequence given the primordial sequence as follows: To sample nucleotide *u* of the descent species *o*, we apply to nucleotide *u* of the primordial sequence the F81 [28] mutation model with the background equilibrium distribution *π*_0_ and the substitution probability *γ*_*o*_.

The generating process for motif-bearing sequences is slightly more complex, since it has to deal both with the generation of the binding site and with the selection process. First, we describe how to sample an alignment without taking into account the selection process. Second, we show how to modify this procedure so that the selection process is considered.

Sample a motif-bearing alignment *X*_*n*_ as follows:

1. Sample the start position of the binding site *ℓ*_*n*_ from the uniform distribution.
2. Sample the primordial sequence. For each position *u* of the sequence outside the binding site, we sample nucleotide ${Y_{n}^{u}}$ from the background equilibrium distribution *π*_0_. For each position *u* of the binding site, we sample nucleotide ${Y_{n}^{u}}$ from the equilibrium distribution $\pi _{u-\ell _{n}+1}$.
3. For each of the descent species *o*∈{1,…,*O*}, sample its sequence $X_{n}^{.,o}$ as follows: For each position *u* of the descent species *o* outside the binding site, apply to nucleotide $X_{n}^{u,o}$ of the primordial sequence the F81 mutation model taking as equilibrium distribution *π*_0_. For each position *u* of the descent species *o* inside the binding site, apply to nucleotide $X_{n}^{u,o}$ of the primordial sequence the F81 mutation model taking as equilibrium distribution $\pi _{u-\ell _{n}+1}$.

Finally, to model the selection process, we introduce the variable *β*. *β* is used to quantify the degree of the binding-affinity bias in the reference species. We assume that a transcription factor binds binding site *B* with a probability proportional to *p*(*B*|*π*)^*β*−1^. As *B* occurs in vivo with probability *p*(*B*|*π*), it occurs in the set of immunoprecipitated sequences with a probability proportional to *p*(*B*|*π*)·*p*(*B*|*π*)^*β*−1^=*p*(*B*|*π*)^*β*^.

We can interpret the meaning of *β* as follows: If *β* is greater than one, low-affinity binding sites are more frequently rejected with respect to *p*(*B*) and high-affinity binding sites are less frequently rejected with respect to *p*(*B*). This leads to an under-representation of low-affinity binding sites and an over-representation of high-affinity binding sites in the ChIP-seq data set, thus modeling a data set that is affected by the binding-affinity bias. If *β* is equal to one, low-affinity binding sites are rejected as frequently as high-affinity binding sites, leading to a representative set of binding sites in the ChIP-seq data set, which is not affected by the binding-affinity bias.

Based on that selection model, sample a motif-bearing alignment that has passed the selection process as follows:

1. Sample a motif-bearing alignment disregarding the selection process following the procedure specified above.
2. Decide whether the alignment is accepted or rejected based on the probability of acceptance of the binding site found at the reference species. If the alignment is rejected, go to step 1.

Thus, we denote (i) the model with *α*=0 and *β*=1 by ${\mathcal {M}^{-}_{-}}$, (ii) the model with with *α*>0 and *β*=1 by ${\mathcal {M}_{-}^{\mathrm {C}}}$, (iii) the model with *α*=0 and *β*>1 by ${\mathcal {M}_{\text {BA}}^{-}}$, and (iv) the model with *α*>0 and $\beta > 1{\mathcal {M}_{\text {BA}}^{\mathrm {C}}}$. ${\mathcal {M}^{-}_{-}}$ can neither handle the contamination bias nor the binding-affinity bias. ${\mathcal {M}_{-}^{\mathrm {C}}}$ can only handle the contamination bias, but not the binding-affinity bias. ${\mathcal {M}_{\text {BA}}^{-}}$ can only handle the binding-affinity bias, but not the contamination bias. And ${\mathcal {M}_{\text {BA}}^{\mathrm {C}}}$ can handle both the contamination bias and the binding-affinity bias.

We call ${\mathcal {M}^{-}_{-}}$, ${\mathcal {M}_{-}^{\mathrm {C}}}$, ${\mathcal {M}_{\text {BA}}^{-}}$, and ${\mathcal {M}_{\text {BA}}^{\mathrm {C}}}$*foreground models*. For modeling the background alignments, we use the model with *α*=1 and *β*=1, which we call *background model* and which we denote by $\mathcal {B}$.

### Measuring classification performance

**Fig. 5:**
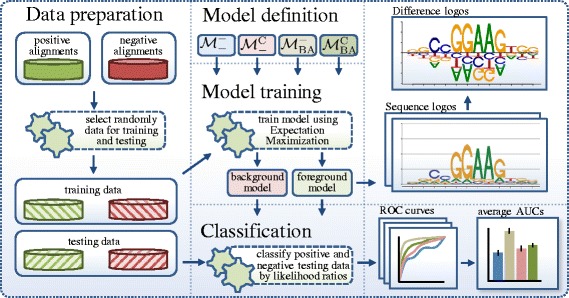
Overview of the workflow presented in this manuscript. In the data preparation step, we randomly compile disjoint training data and testing data each with positive alignments and negative alignments for each of the transcription factors CTCF, GABP, NRSF, SRF, and STAT1. In the model training step, we train each of the four presented foreground models as well as a background model by expectation maximization with 150 restarts. We choose the foreground model and the background model with maximum likelihood, classify the testing data using a likelihood-ratio classifier, and extract different characteristics such as the ROC curve, the PR curve, the inverse temperature, and the inferred motif. We repeat the described procedure 100 times and calculate mean values and standard errors for several quantities such as the areas under the ROC curves or the PR curves

For measuring the classification performance of the four models ${\mathcal {M}^{-}_{-}}$, ${\mathcal {M}_{\text {BA}}^{-}}$, ${\mathcal {M}_{-}^{\mathrm {C}}}$, and ${\mathcal {M}_{\text {BA}}^{\mathrm {C}}}$ we perform stratified repeated random sub-sampling validation as illustrated in Fig. 5 using data sets of the five human transcription factors CTCF, GABP, NRSF, SRF, and STAT1 that have been used for benchmarking the phylogenetic footprinting program *MotEvo* [25].

In step 1, we generate two training sets and two disjoint test sets for each of the five transcription factors as follows. We randomly select 200 alignments from the set of alignments (Table 2) of a particular transcription factor as positive training set, and we choose the set of the remaining alignments as positive test set. We randomly select 500 alignments from the set of alignments of the four remaining transcription factors as negative training set and another disjoint set of 500 alignments as negative test set.

In step 2, we train a foreground model (${\mathcal {M}^{-}_{-}}$, ${\mathcal {M}_{\text {BA}}^{-}}$, ${\mathcal {M}_{-}^{\mathrm {C}}}$, or ${\mathcal {M}_{\text {BA}}^{\mathrm {C}}}$) on the positive training set and a background model ($\mathcal {B}$) on the negative training set by expectation maximization [29] using a numerical optimization procedure in the maximization step.

We restart the expectation maximization algorithm, which is deterministic for a given data set and a given initialization, 150 times with different initializations and choose the foreground model and the background model with the maximum likelihood on the positive training data and the negative training data, respectively, for classification. We use a likelihood-ratio classifier of the two chosen foreground and background models, apply this classifier to the disjoint positive and negative test sets, and calculate the receiver operating characteristics curve, the precision recall curve, and the area under both curves as measures of classification performance.

We repeat both steps 100 times and determine (i) the mean area under the receiver operating characteristic curve and its standard error and (ii) the mean area under the precision recall curve and its standard error.

### Data

The data used in this work originate from human ChIP-seq data of the five human transcription factors CTCF, GABP, NRSF, SRF, and STAT1, where the ChIP-seq data for GABP and SRF published in [10] are available from the QuEST web page [30], and the ChIP-seq data for CTCF, NRSF, and STAT1 published in [24] are available from the SISSRs web page [31]. All five data sets have been filtered for high-quality reads and mapped to a reference genome [10, 24], and peak calling has been performed by MACS [32]. Peaks have been extended or cropped to 400 bp, binding regions that potentially comprise more than one of the five transcription factors have been removed, and the 900 binding regions with the highest MACS score have been retained [25]. Orthologous regions from mouse, dog, cow, monkey, horse, and opossum have been extracted from the UCSC database [33], multiple alignments of these orthologous regions have been obtained using T-Coffee [34], and these multiple alignments are kindly provided by [25].

To prepare ungapped alignments from these gapped data sets of the five transcription factors CTCF, GABP, NRSF, SRF, and STAT1, we perform the following three steps. (i) Remove the species that cause the highest number of gaps in all alignments. Accordingly, we remove mouse and opossum and keep orthologous regions from human, monkey, cow, dog, and horse. (ii) Remove all columns in each of the alignments that contain at least one gap to obtain ungapped alignments. (iii) Remove all ungapped alignments that are shorter than 21 bp, which is the length of the longest motif (NRSF) in the performed studies. Table 2 shows details about the resulting data. All data are available as Additional file 2.

### Visualizing motif differences with DiffLogo

We used the R package *DiffLogo* [35] to depict the differences between the predicted motifs of the models ${\mathcal {M}^{-}_{-}}$, ${\mathcal {M}_{\text {BA}}^{-}}$, ${\mathcal {M}_{-}^{\mathrm {C}}}$, and ${\mathcal {M}_{\text {BA}}^{\mathrm {C}}}$. DiffLogo is an open source software that is capable of depicting the differences between multiple motifs [35]. This is realized by visualizing all pair-wise differences in an *N*×*N*–grid with an empty diagonal. Each entry in the grid is called *difference logo*. The degree of difference of two motifs is calculated by the sum of all stack heights in the corresponding difference logo and is indicated by the background color from red (most dissimilar among all motif pairs) to green (most similar among all motif pairs). The individual sequence logos of the motifs are shown above the table.

A single difference logo depicts the position-specific differences between the base distributions of two sequence motifs. Differences are visualized using a stack of bases for each motif position. The height of each base stack is calculated by the Jensen-Shannon divergence, which is proportional to the degree of base distribution dissimilarity. The Jensen-Shannon divergence is zero if both base distributions are identical, increases with increasing difference of the two base distributions, and reaches a maximum of 2 bit if the two base distributions are maximally different, i.e., if two bases occur only in one of the two motifs each with a probability of 1/2 and the other two bases occur only in the other motif each with a probability of 1/2. The height of each base within a stack is given by the difference of abundance. Thus, the height of a base is proportional to the degree of differential symbol abundance. Bases with a positive height indicate a gain of abundance and bases with a negative height indicate a loss of abundance. The stack height in the positive direction must be equal to the stack height in the negative direction, because the sum of base abundance gain must be equal to the sum of base abundance loss.

## Additional files

Additional file 1 In section 1, *Modeling the binding-affinity bias*, we describe how to determine the likelihood of non-motif-bearing and motif-bearing alignments modeling the contamination bias and the binding-affinity bias. In section 2, *Example interpretation of difference logos*, we give an exemplary interpretation of some difference logos. Section 3, *Supplementary Figures*, contains supplementary Figures S1-S18. Section 4, *Supplementary Tables*, contains supplementary Tables S1-S10. (PDF 3492 kb)

Additional file 2 Sequence data. This archive contains data files of gap-free alignments of the ChIP-seq positive regions for each of the transcription factors CTCF, GABP, NRSF, SRF, and STAT1 in FASTA format. (ZIP 645 kb)
