# Supplementary material for: Detecting and correcting the binding-affinity bias in ChIP-seq data using inter-species information
Source: BMC Genomics. 2016 May 10;17:347. doi: 10.1186/s12864-016-2682-6 (PMC4862171; doi:10.1186/s12864-016-2682-6)
Supplement: Additional file 1 — In section 1, Modeling the binding-affinity bias, we describe how to determine the likelihood of non-motif-bearing and motif-bearing alignments modeling the contamination bias and the binding-affinity bias. In section 2, Example interpretation of difference logos, we give an exemplary interpretation of some difference logos. Section 3, Supplementary Figures, contains supplementary Figures S1-S18. Section 4, Supplementary Tables, contains supplementary Tables S1-S10. (PDF 3492 kb) [file 12864_2016_2682_MOESM1_ESM.pdf]

1 Modeling the binding-affinity bias

In this section we describe the probabilistic model for modeling the binding-affinity bias. We define the model in mathematical terms by providing the likelihood function. We use the notation from the manuscript.

Following the data-generating process described in the manuscript, the probability that the model generates an alignment X_n can be written as

$$\begin{aligned} p(X_n|\theta) &= p(X_n|M_n = 0, \theta) \cdot p(M_n = 0, \theta) + p(X_n|M_n = 1, \theta) \cdot p(M_n = 1, \theta) \\ &= p(X_n|M_n = 0, \theta) \cdot \alpha + p(X_n|M_n = 1, \theta) \cdot (1 - \alpha) \end{aligned}$$

To complete the model, we need to specify the probability for non-motif-bearing alignments $p(X_n|M_n = 0, \theta)$ and that for motif-bearing alignments $p(X_n|M_n = 1, \theta)$.

Likelihood of a non-motif-bearing alignment Looking at the description of the generating process for non-motif-bearing alignments we get

$$p(X_n|M_n = 0, \theta) = \sum_{Y_n \in \mathcal{A}^{L_n}} p(Y_n|M_n = 0, \theta) \prod_{o=1}^{O} p(X_n^{:,o}|Y_n, M_n = 0, \theta).$$

Note that given θ and $M_n = 0$, each single nucleotide alignment is independent of any other single nucleotide alignment. Thus, the likelihood can be expressed as

$$p(X_n|M_n = 0, \theta) = \prod_{u=1}^{L_n} \sum_{Y_n^u \in \mathcal{A}} p(Y_n^u|M_n = 0, \theta) \prod_{o=1}^{O} p(X_n^{u,o}|Y_n^u, M_n = 0, \theta).$$

Here we denote $p(Y_n^u|M_n = 0, \theta)$ and $p(X_n^{u,o}|Y_n^u, M_n = 0, \theta)$ by parameters

$$\begin{aligned} p(Y_n^u|M_n = 0, \theta) &= \pi_0^{Y_n^u} \\ p(X_n^{u,o}|Y_n^u, M_n = 0, \theta) &= \gamma_o \cdot \pi_0^{X_n^{u,o}} + (1 - \gamma_o) \cdot \delta_{X_n^{u,o} = Y_n^u} \end{aligned}$$

according to the F81 model, where the base distribution of each position of the background sequence is denoted by π_0, the probability of a nucleotide a in the background sequence is denoted by π_0^a, and the substitution probability from the primordial species to species o is denoted by γ_o.

Likelihood of a motif-bearing alignment In the data generating process for motif-bearing alignments we sample alignments until one of them is accepted. Mapping this into a likelihood requires the usage of the *Felsenstein's pulley principle* [1], that allows us to select any particular species as the root of the tree. In this case it will come handy to select the reference species as the root. Thus, the likelihood can be expressed as

$$\begin{aligned} p(X_n|M_n = 1, \theta) = \sum_{\ell_n=1}^{L_n-W+1} & p(X_n^{:,1}|M_n = 1, \ell_n) \cdot \sum_{Y_n \in \mathcal{A}^{L_n}} p(Y_n|X_n^{:,1}, M_n = 1, \ell_n) \cdot \\ & \prod_{o=2}^{O} p(X_n^{:,o}|Y_n, M_n = 1, \ell_n) p(\ell_n|M_n = 1, \theta), \end{aligned}$$

where the base distributions of the positions $1, \ldots, W$ of the binding sites are denoted by $\pi_1, \ldots, \pi_W$ and the probability of a nucleotide a in the binding site at position w is denoted by π_w^a.

Given π, $\ell_n \in \{1, \ldots, L_n - W + 1\}$, and $M_n = 1$, each single nucleotide alignment is independent of any other single nucleotide alignment, and we obtain

$$p(X_n | M_n = 1, \theta) = \sum_{\ell_n=1}^{L_n-W+1} \prod_{u=1}^{L_n} p(X_n^{u,1} | M_n = 1, \ell_n) \cdot \sum_{Y_n^u \in \mathcal{A}} p(Y_n | X_n^{u,1}, M_n = 1, \ell_n) \cdot$$
$$\prod_{o=2}^{O} p(X_n^{u,o} | Y_n, M_n = 1, \ell_n) p(\ell_n | M_n = 1, \theta).$$

We need to determine the probability of a particular nucleotide in a specific position of the reference species after selection, that is $p(X_n^{u,1} | M_n = 1, \ell_n)$. On one hand, notice that selection does not affect the probability distribution of those nucleotides outside the binding site. Thus, for $u < \ell_n$ or $u \geq \ell_n + W$ we have that $p(X_n^{u,1} = a | M_n = 1, \ell_n) = \pi_0^a$. On the other hand, for nucleotides in the binding site, the distribution after filtering is $p(X_n^{u,1} = a | M_n = 1, \ell_n) \propto (\pi_{u-\ell_n+1}^a)^\beta$. Thus, $p(X_n^{u,1} = a | M_n = 1, \ell_n) = \frac{(\pi_{u-\ell_n+1}^a)^\beta}{\sum_{b \in \mathcal{A}} (\pi_{u-\ell_n+1}^b)^\beta}$.

The probabilities for the nucleotides in the ancestral sequence and in the non-reference species are given by the F81 model. In particular, for the ancestral sequence

$$p(Y_n = a | X_n^{u,1} = b, M_n = 1, \ell_n) = \begin{cases} \gamma_1 \cdot \pi_0^a + (1 - \gamma_1) \cdot \delta_{a=b} & \text{, if } u < \ell_n \text{ or } u \geq \ell_n + W \\ \gamma_1 \cdot \pi_{u-\ell_n+1}^a + (1 - \gamma_1) \cdot \delta_{a=b} & \text{, if } \ell_n \leq u < \ell_n + W \end{cases}$$

and for the non reference species

$$p(X_n^{u,o} = a | Y_n = b, M_n = 1, \ell_n) = \begin{cases} \gamma_o \cdot \pi_0^a + (1 - \gamma_o) \cdot \delta_{a=b} & \text{, if } u < \ell_n \text{ or } u \geq \ell_n + W \\ \gamma_o \cdot \pi_{u-\ell_n+1}^a + (1 - \gamma_o) \cdot \delta_{a=b} & \text{, if } \ell_n \leq u < \ell_n + W \end{cases}$$

Finally, since we assume binding sites to be uniformly distributed, we have that $p(\ell_n | M_n = 1, \theta) = \frac{1}{L_n - W + 1}$. This completes the specification of the likelihood function.

2 Example interpretation of difference logos

We give an exemplary interpretation of the difference logos in row 1 of Figure S5. Here, the motif inferred by $\mathcal{M}_{-}^{-}$ (column 1) is used as the reference and is compared to the motifs inferred by $\mathcal{M}_{\mathrm{BA}}^{-}$ (column 2), $\mathcal{M}_{-}^{\mathrm{C}}$ (column 3), and $\mathcal{M}_{\mathrm{BA}}^{\mathrm{C}}$ (column 4). As indicated by the background colors, the smallest difference can be observed for $\mathcal{M}_{-}^{\mathrm{C}}$ and the largest difference can be observed for $\mathcal{M}_{\mathrm{BA}}^{-}$. No difference logo shows notable differences at motif positions 1 to 3 and 17 to 21.

In the difference logos for $\mathcal{M}_{\mathrm{BA}}^{-}$ and $\mathcal{M}_{\mathrm{BA}}^{\mathrm{C}}$ we observe a decrease of the most abundant bases (below the abscissa) and a gain of the remaining bases (above the abscissa). In contrast, the difference logo for $\mathcal{M}_{-}^{\mathrm{C}}$ shows mainly a gain of cytosine at motif position 6 and a loss of the remaining bases. All other motif positions show much smaller Jensen–Shannon divergences. We can observe the opposite behaviour in the difference logo for $\mathcal{M}_{\mathrm{BA}}^{-}$ at motif position 6. This discrepancy seems to be compensated in the difference logo for $\mathcal{M}_{\mathrm{BA}}^{\mathrm{C}}$. This compensation cannot be observed for the other motif positions. Here, the differences are similar to these in the difference logo $\mathcal{M}_{\mathrm{BA}}^{-}$.

3 Supplementary figures

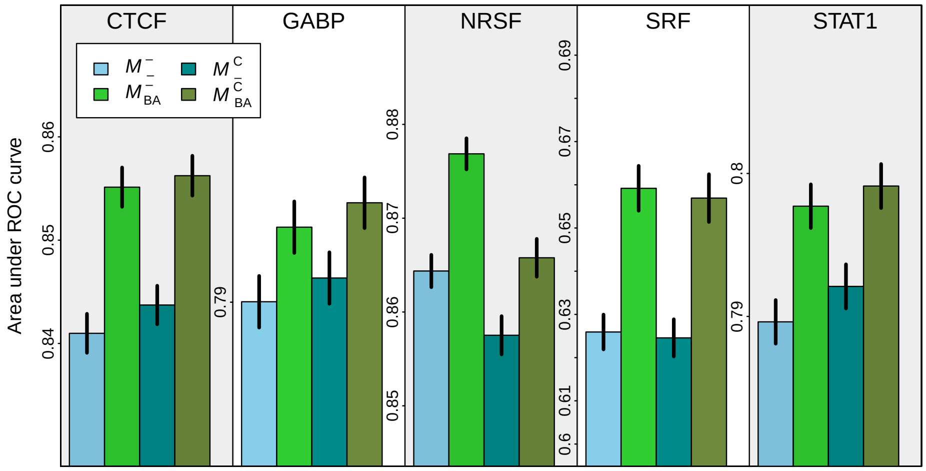

Figure S1: Classification performance measured by the area under the receiver operating characteristic curve. We compute the areas under the receiver operating characteristic curves for each of the four classifiers consisting of foreground models $\mathcal{M}_{-}^{-}$, $\mathcal{M}_{\mathrm{BA}}^{-}$, $\mathcal{M}_{-}^{\mathrm{C}}$, and $\mathcal{M}_{\mathrm{BA}}^{\mathrm{C}}$ and background model $\mathcal{B}$ for each of the five data sets corresponding to the transcription factors CTCF, GABP, NRSF, SRF, and STAT1. We perform a stratified repeated random sub-sampling validation and show the means and their standard errors for each of the four models and each of the five data sets. We find that $\mathcal{M}_{\mathrm{BA}}^{\mathrm{C}}$ yields a significantly higher classification performance than $\mathcal{M}_{-}^{\mathrm{C}}$ and that $\mathcal{M}_{\mathrm{BA}}^{-}$ yields a significantly higher classification performance than $\mathcal{M}_{-}^{-}$ for all five data sets (**Supplementary Table S2**), stating that despite the oversimplified assumption of the Boltzmann distribution the new models that take into account the binding-affinity bias are always more realistic than the traditional models that neglect this bias.

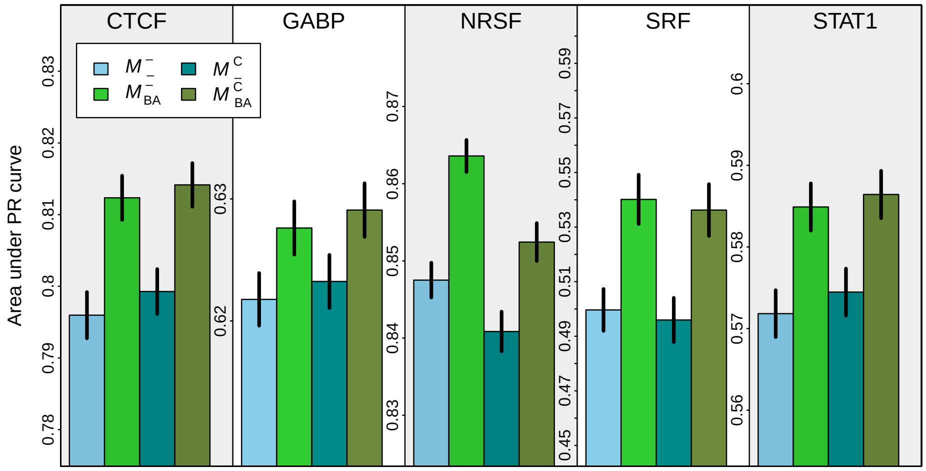

Figure S2: Classification performance measured by the area under the precision recall curve. We compute the area under the precision recall curves for each of the four classifiers consisting of foreground models $\mathcal{M}^{-}_{-}$, $\mathcal{M}^{-}_{\mathrm{BA}}$, $\mathcal{M}^{\mathrm{C}}_{-}$, and $\mathcal{M}^{\mathrm{C}}_{\mathrm{BA}}$ and background model $\mathcal{B}$ for each of the five data sets corresponding to the transcription factors CTCF, GABP, NRSF, SRF, and STAT1. We perform a stratified repeated random sub-sampling validation and show the means and their standard errors for each of the four models and each of the five data sets. We find that $\mathcal{M}^{\mathrm{C}}_{\mathrm{BA}}$ yields a significantly higher classification performance than $\mathcal{M}^{\mathrm{C}}_{-}$ and that $\mathcal{M}^{-}_{\mathrm{BA}}$ yields a significantly higher classification performance than $\mathcal{M}^{-}_{-}$ for all five data sets (**Supplementary Table S3**), stating that despite the oversimplified assumption of the Boltzmann distribution the new models that take into account the binding-affinity bias are always more realistic than the traditional models that neglect this bias.

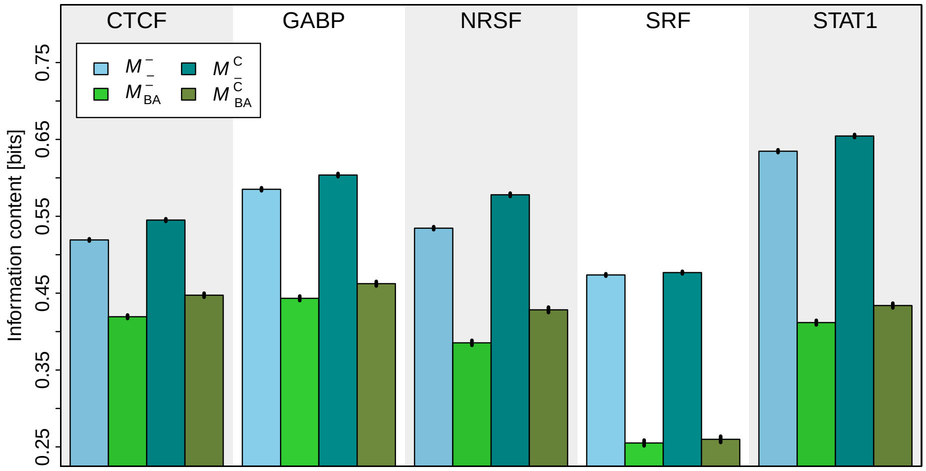

Figure S3: Information contents of the inferred motifs. We compute the information contents for each of the inferred motifs from $\mathcal{M}^{-}_{-}$, $\mathcal{M}^{-}_{\mathrm{BA}}$, $\mathcal{M}^{\mathrm{C}}_{-}$, and $\mathcal{M}^{\mathrm{C}}_{\mathrm{BA}}$ for the data sets of each of the transcription factors CTCF, GABP, NRSF, SRF, and STAT1. We perform a stratified repeated random sub-sampling validation and show the means and their standard errors for each of the four models and each of the five data sets. The information contents of motifs inferred by $\mathcal{M}^{-}_{\mathrm{BA}}$ and $\mathcal{M}^{\mathrm{C}}_{\mathrm{BA}}$ are significantly smaller than the information contents of motifs inferred by $\mathcal{M}^{-}_{-}$ and $\mathcal{M}^{\mathrm{C}}_{-}$ in each of the five data sets (**Supplementary Table S4**), indicating an enrichment of high-affinity binding sites in all cases. In addition, the information contents of motifs inferred by $\mathcal{M}^{-}_{-}$ are higher than the information contents of motifs inferred by $\mathcal{M}^{\mathrm{C}}_{\mathrm{BA}}$ in all cases, indicating that the superposition of the contamination bias and the binding-affinity bias leads to a sharpening of the motifs.

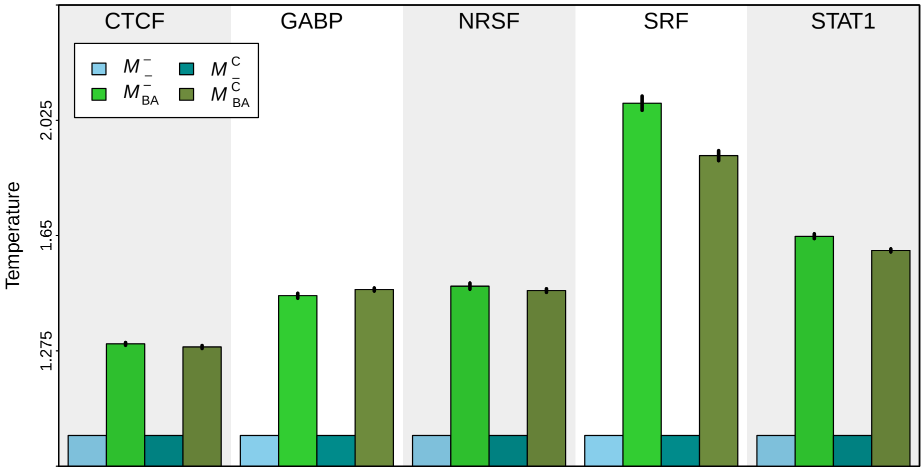

Figure S4: Inverse temperatures of $\mathcal{M}_{-}^{-}$,$\mathcal{M}_{-}^{\mathrm{C}}$,$\mathcal{M}_{\mathrm{BA}}^{-}$, and $\mathcal{M}_{\mathrm{BA}}^{\mathrm{C}}$. We plot the inverse temperatures β for each of the learned models $\mathcal{M}_{\mathrm{BA}}^{-}$ and $\mathcal{M}_{\mathrm{BA}}^{\mathrm{C}}$ for the data sets of each of the transcription factors CTCF, GABP, NRSF, SRF, and STAT1. We perform a stratified repeated random sub-sampling validation and show the means of β and their standard errors for the models $\mathcal{M}_{\mathrm{BA}}^{-}$ and $\mathcal{M}_{\mathrm{BA}}^{\mathrm{C}}$ for each of the five data sets together with the values of $\beta = 1$ for $\mathcal{M}_{-}^{-}$ and $\mathcal{M}_{-}^{\mathrm{C}}$. We find that β is significantly greater than 1 in both cases (**Supplementary Table S5**), indicating an enrichment of high-affinity binding sites in all five data sets.

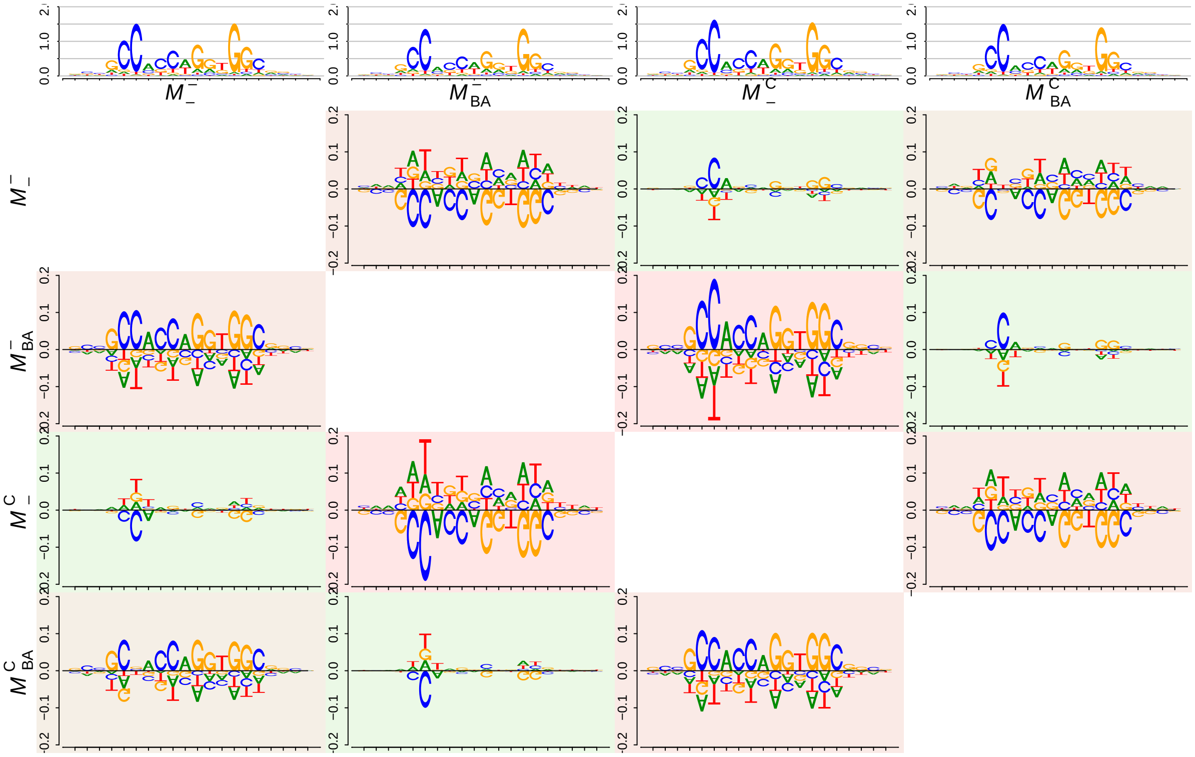

Figure S5: DiffLogo Table of motifs inferred by $\mathcal{M}_{-}^{-}$,$\mathcal{M}_{-}^{\mathrm{C}}$,$\mathcal{M}_{\mathrm{BA}}^{-}$, and $\mathcal{M}_{\mathrm{BA}}^{\mathrm{C}}$ for the transcription factor CTCF.

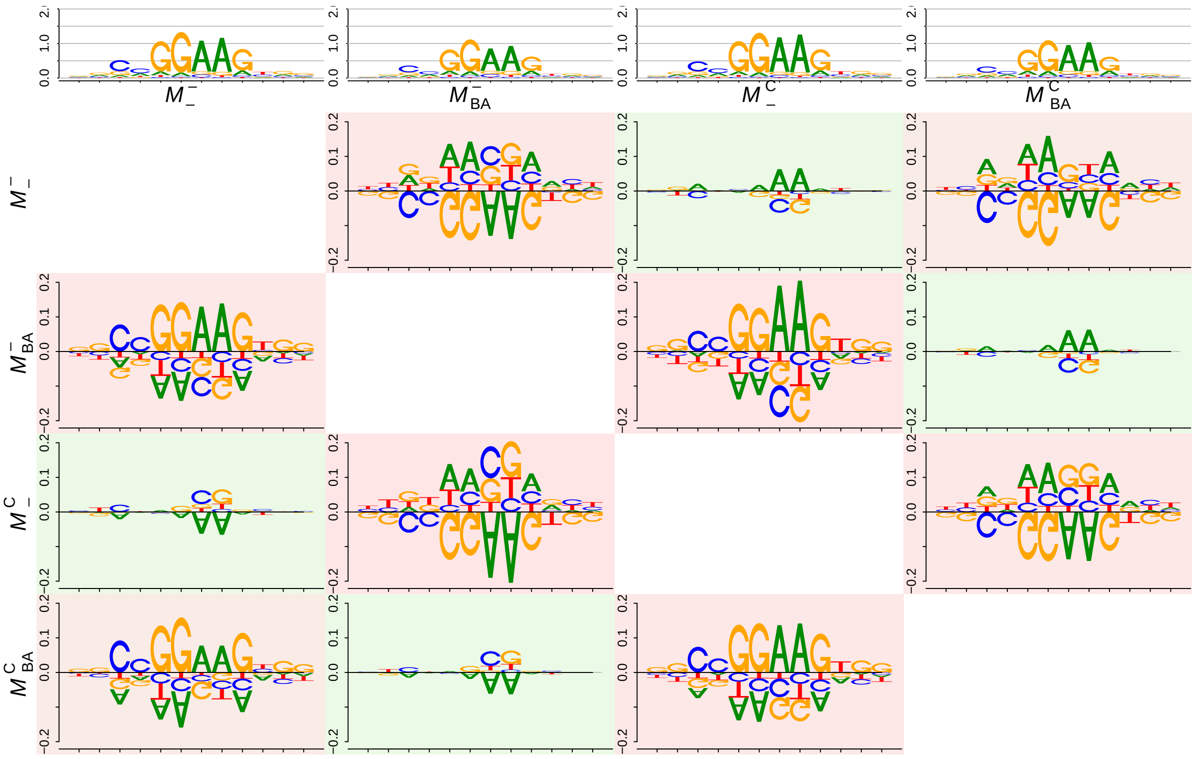

Figure S6: DiffLogo Table of motifs inferred by $\mathcal{M}_{-}^{-}$,$\mathcal{M}_{-}^{\mathrm{C}}$,$\mathcal{M}_{\mathrm{BA}}^{-}$, and $\mathcal{M}_{\mathrm{BA}}^{\mathrm{C}}$ for the transcription factor GABP.

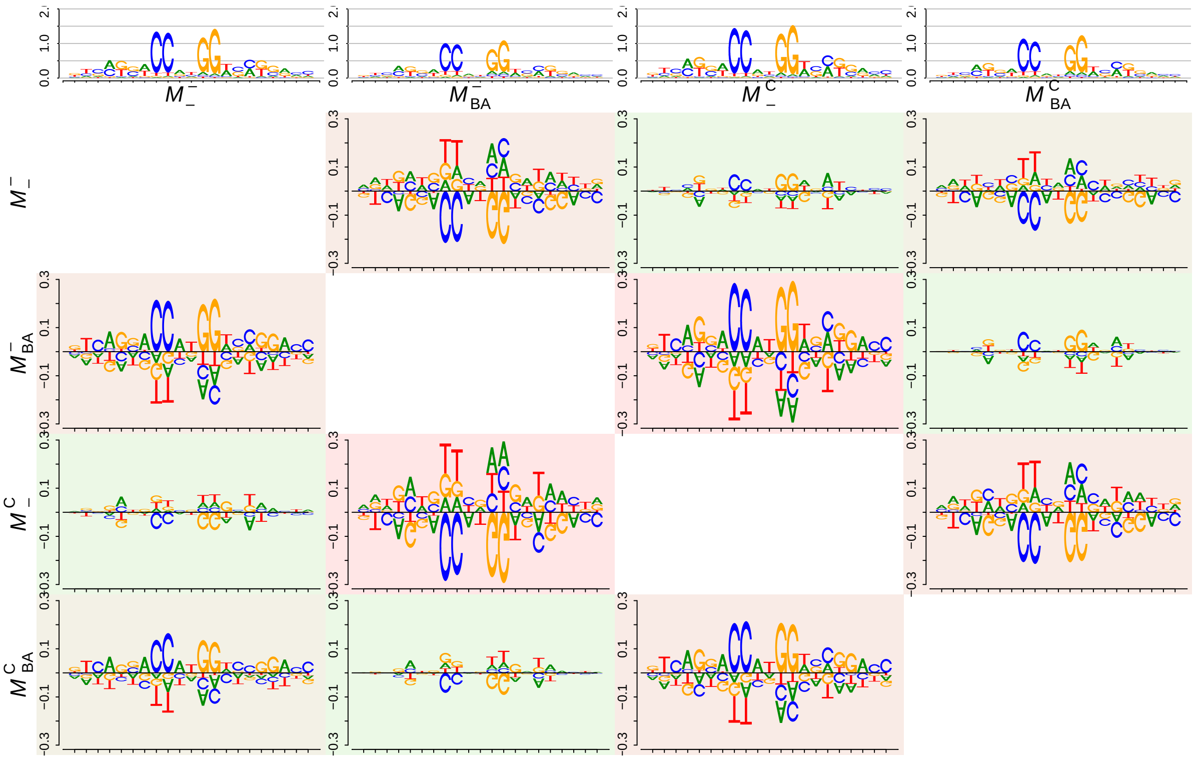

Figure S7: DiffLogo Table of motifs inferred by $\mathcal{M}_{-}^{-}$,$\mathcal{M}_{-}^{\mathrm{C}}$,$\mathcal{M}_{\mathrm{BA}}^{-}$, and $\mathcal{M}_{\mathrm{BA}}^{\mathrm{C}}$ for the transcription factor NRSF.

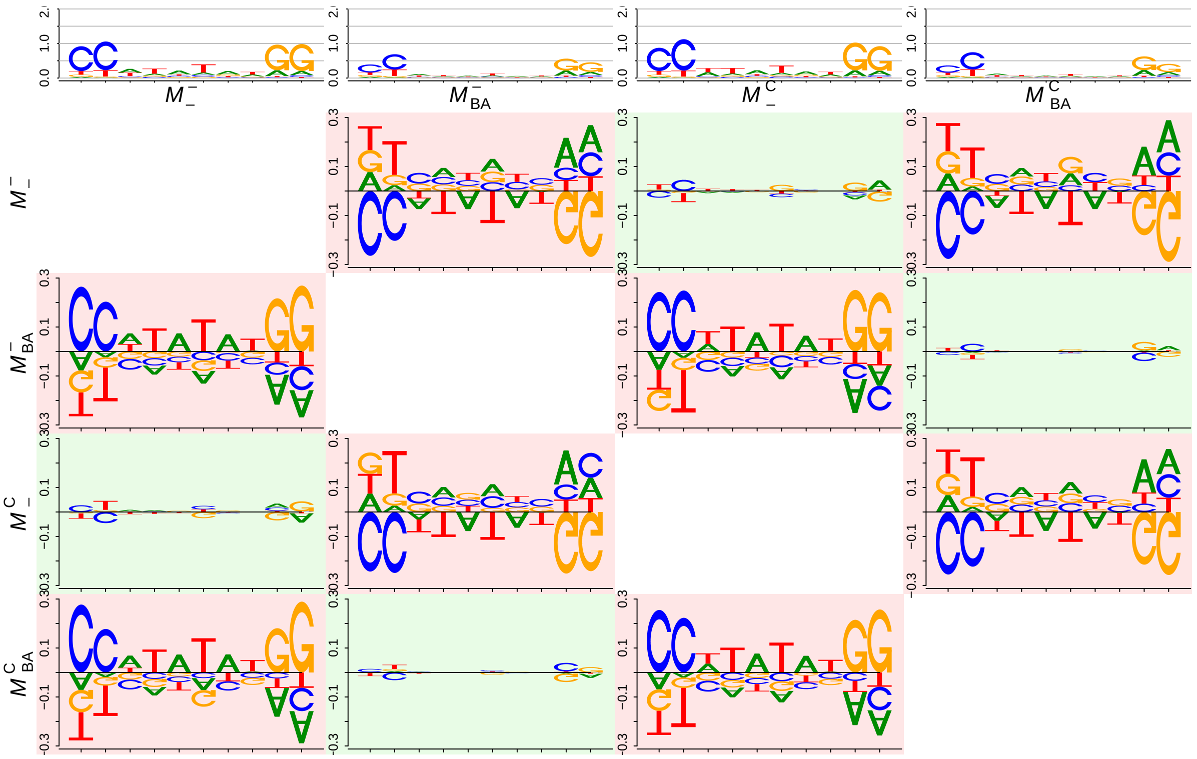

Figure S8: DiffLogo Table of motifs inferred by $\mathcal{M}_{-}^{-}$,$\mathcal{M}_{-}^{\mathrm{C}}$,$\mathcal{M}_{\mathrm{BA}}^{-}$, and $\mathcal{M}_{\mathrm{BA}}^{\mathrm{C}}$ for the transcription factor SRF.

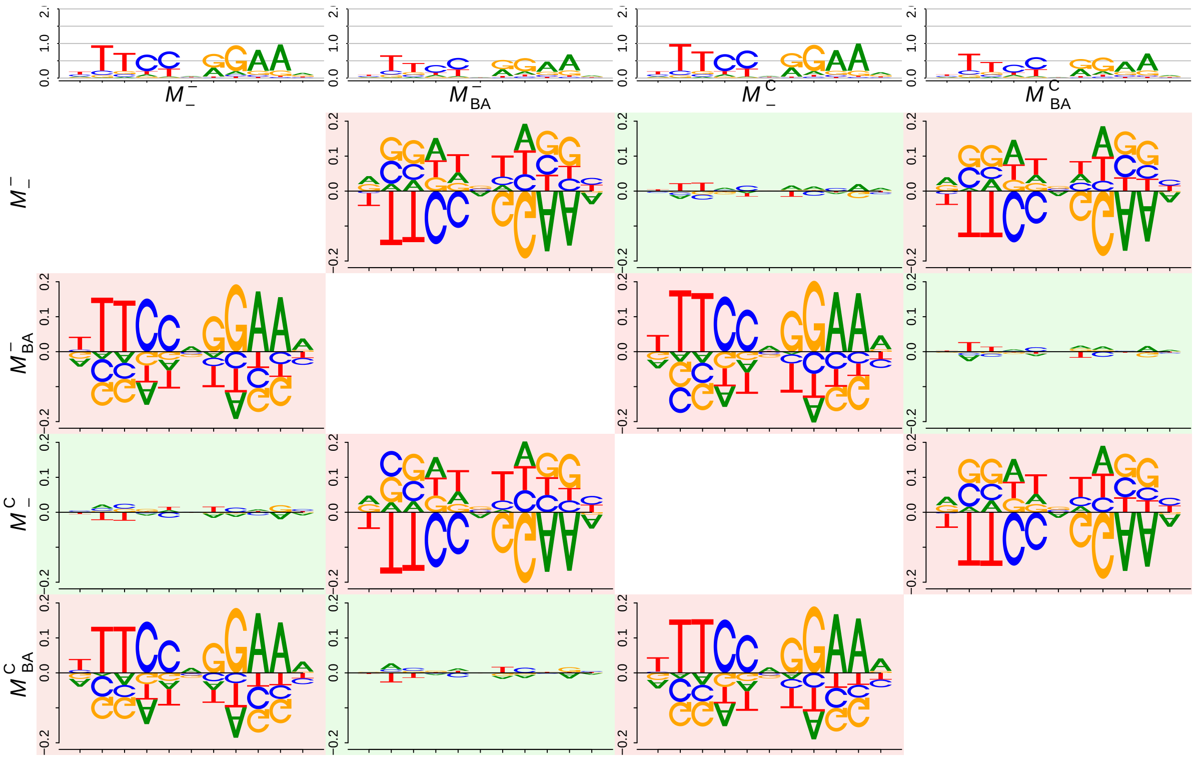

Figure S9: DiffLogo Table of motifs inferred by $\mathcal{M}_{-}^{-}$,$\mathcal{M}_{-}^{\mathrm{C}}$,$\mathcal{M}_{\mathrm{BA}}^{-}$, and $\mathcal{M}_{\mathrm{BA}}^{\mathrm{C}}$ for the transcription factor STAT1.

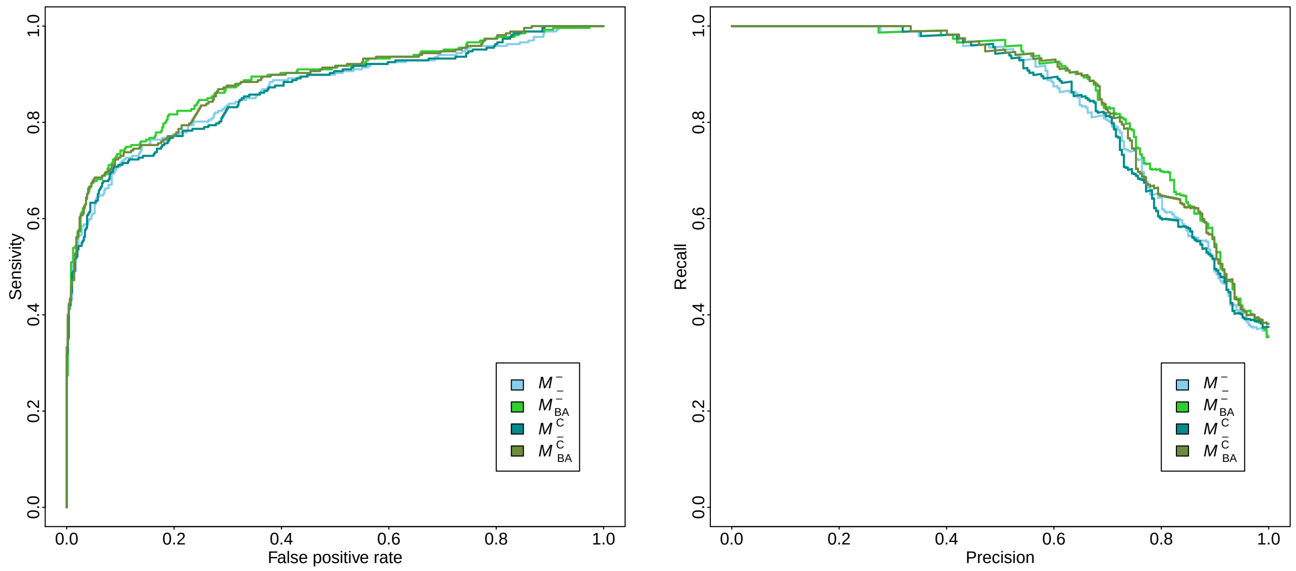

Figure S10: Receiver operating characteristics curves (left panel) and precision recall curves (right panel) for each of the four classifiers consisting of foreground models $\mathcal{M}^{-}_{-}$, $\mathcal{M}^{-}_{\mathrm{BA}}$, $\mathcal{M}^{\mathrm{C}}_{-}$, and $\mathcal{M}^{\mathrm{C}}_{\mathrm{BA}}$ and background model $\mathcal{B}$ for one run result on the CTCF data set, calculated using 100 fold stratified repeated random sub-sampling validation.

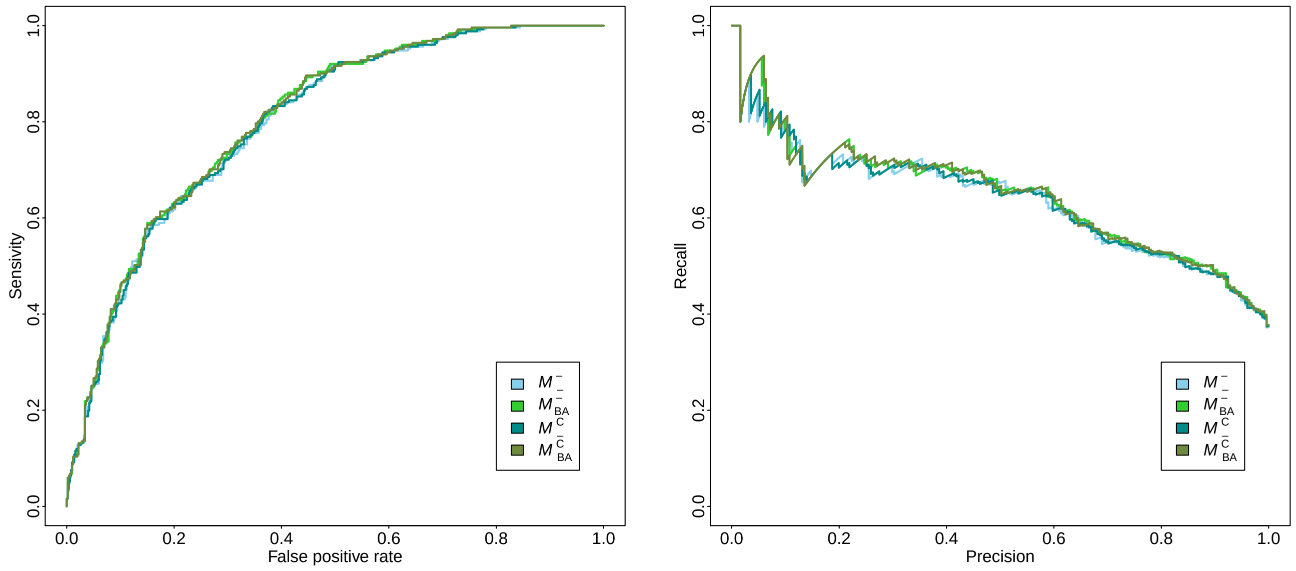

Figure S11: Receiver operating characteristics curves (left panel) and precision recall curves (right panel) for each of the four classifiers consisting of foreground models $\mathcal{M}^{-}_{-}$, $\mathcal{M}^{-}_{\mathrm{BA}}$, $\mathcal{M}^{\mathrm{C}}_{-}$, and $\mathcal{M}^{\mathrm{C}}_{\mathrm{BA}}$ and background model $\mathcal{B}$ for one run result on the GABP data set, calculated using 100 fold stratified repeated random sub-sampling validation.

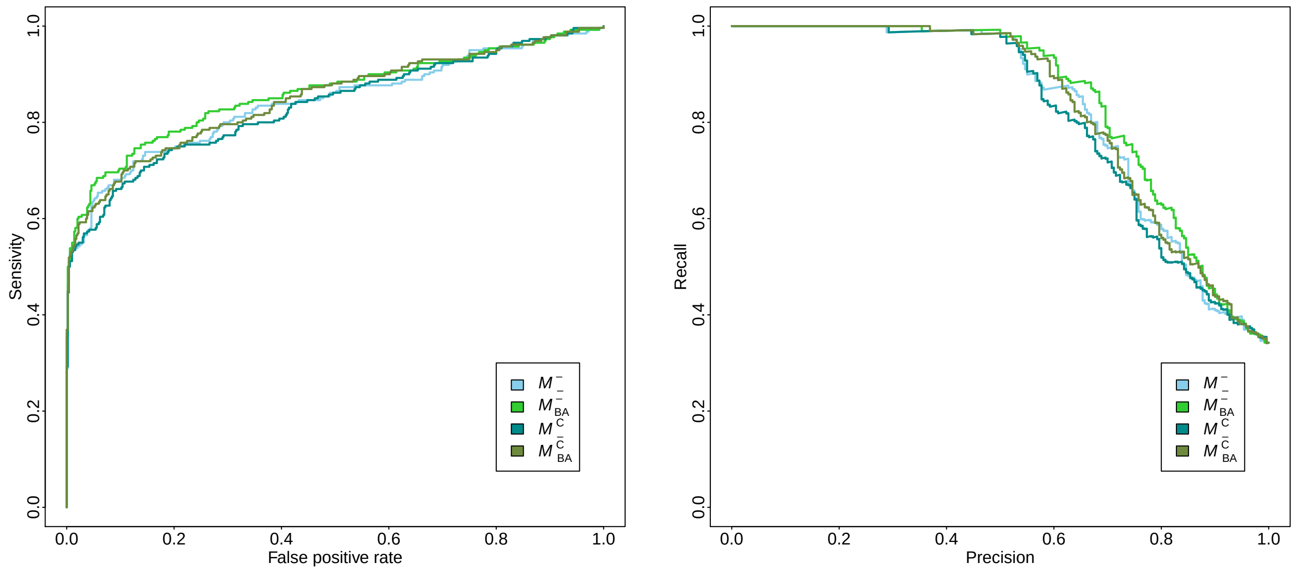

Figure S12: Receiver operating characteristics curves (left panel) and precision recall curves (right panel) for each of the four classifiers consisting of foreground models $\mathcal{M}_{-}^{-}$, $\mathcal{M}_{\mathrm{BA}}^{-}$, $\mathcal{M}_{-}^{\mathrm{C}}$, and $\mathcal{M}_{\mathrm{BA}}^{\mathrm{C}}$ and background model $\mathcal{B}$ for one run result on the NRSF data set, calculated using 100 fold stratified repeated random sub-sampling validation.

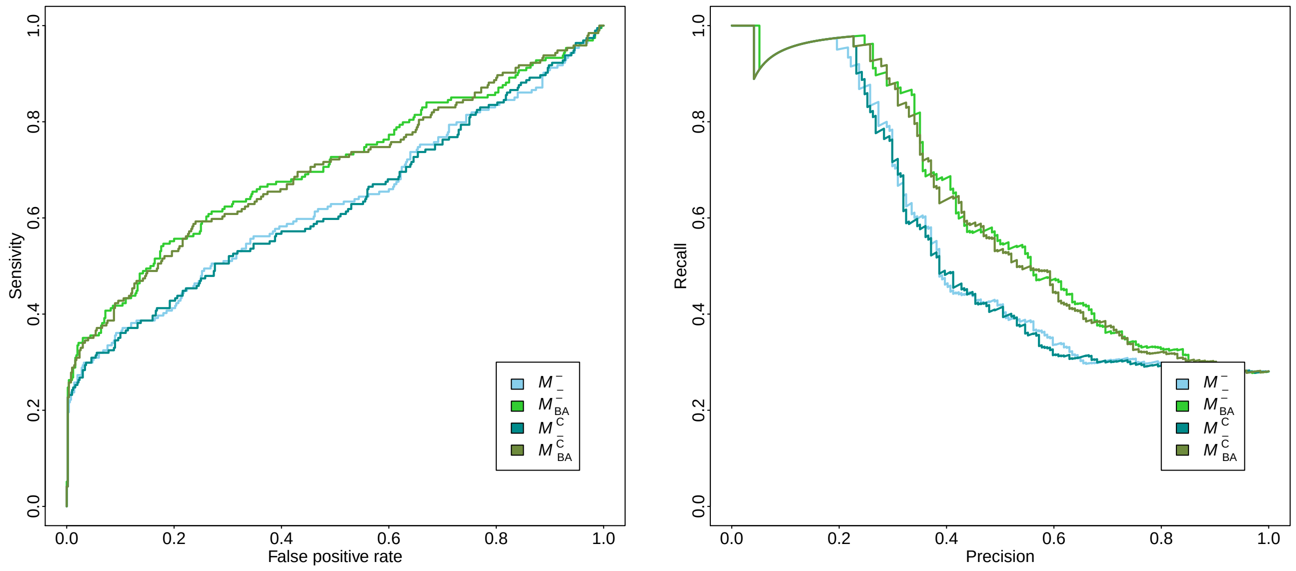

Figure S13: Receiver operating characteristics curves (left panel) and precision recall curves (right panel) for each of the four classifiers consisting of foreground models $\mathcal{M}_{-}^{-}$, $\mathcal{M}_{\mathrm{BA}}^{-}$, $\mathcal{M}_{-}^{\mathrm{C}}$, and $\mathcal{M}_{\mathrm{BA}}^{\mathrm{C}}$ and background model $\mathcal{B}$ for one run result on the SRF data set, calculated using 100 fold stratified repeated random sub-sampling validation.

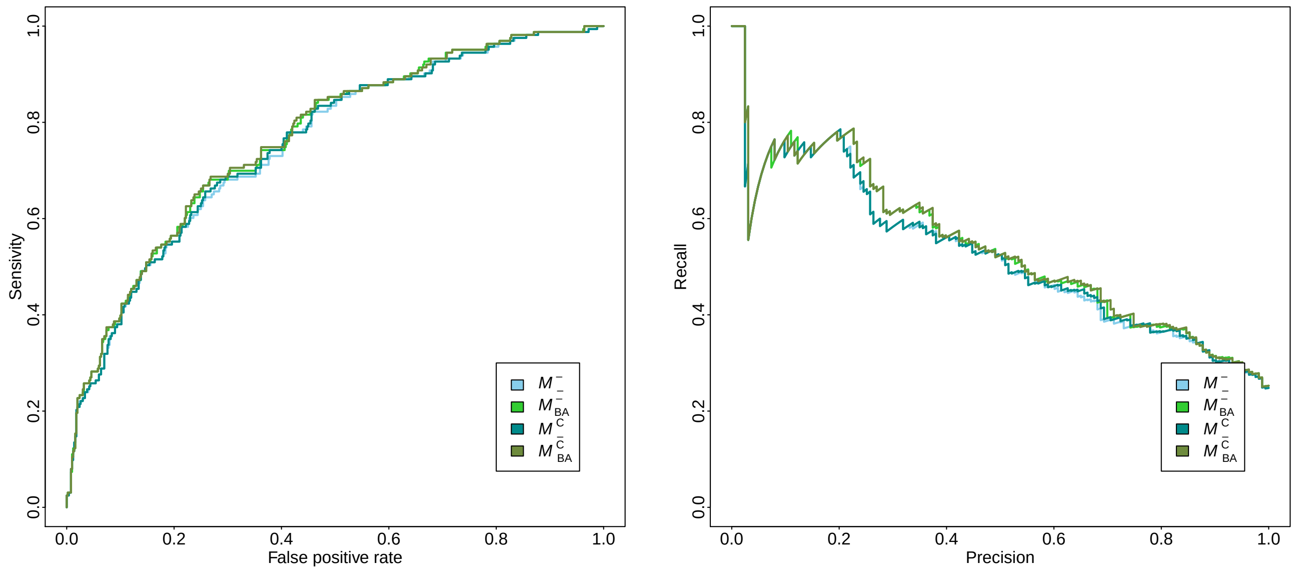

Figure S14: Receiver operating characteristics curves (left panel) and precision recall curves (right panel) for each of the four classifiers consisting of foreground models $\mathcal{M}_{-}^{-}$, $\mathcal{M}_{\mathrm{BA}}^{-}$, $\mathcal{M}_{-}^{\mathrm{C}}$, and $\mathcal{M}_{\mathrm{BA}}^{\mathrm{C}}$ and background model $\mathcal{B}$ for one run result on the STAT1 data set, calculated using 100 fold stratified repeated random sub-sampling validation.

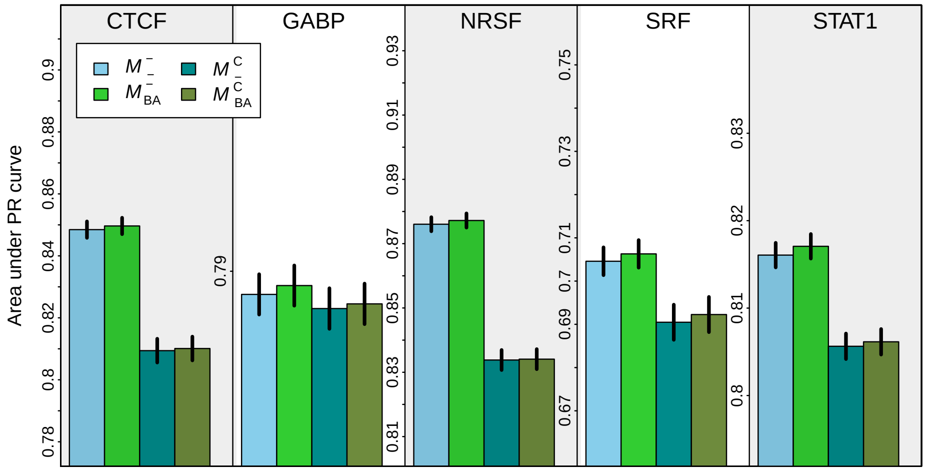

Figure S15: Classification performance measured by the area under the receiver operating characteristic curve for pair-wise alignments of human and monkey. We compute the areas under the receiver operating characteristic curves for each of the four classifiers consisting of foreground models $\mathcal{M}_{-}^{-}$, $\mathcal{M}_{\mathrm{BA}}^{-}$, $\mathcal{M}_{-}^{\mathrm{C}}$, and $\mathcal{M}_{\mathrm{BA}}^{\mathrm{C}}$ and background model $\mathcal{B}$ for each of the five data sets corresponding to the transcription factors CTCF, GABP, NRSF, SRF, and STAT1. We perform a stratified repeated random sub-sampling validation and show the means and their standard errors for each of the four models and each of the five data sets. The classification performance decreases in particular for the models with contamination bias ($\mathcal{M}_{-}^{\mathrm{C}}$ and $\mathcal{M}_{\mathrm{BA}}^{\mathrm{C}}$) compared to the results achieved for five species (see **Figure S1**). We find that $\mathcal{M}_{\mathrm{BA}}^{\mathrm{C}}$ yields a higher classification performance than $\mathcal{M}_{-}^{\mathrm{C}}$ and that $\mathcal{M}_{\mathrm{BA}}^{-}$ yields a higher classification performance than $\mathcal{M}_{-}^{-}$ for all five data sets which we find also in case of five species.

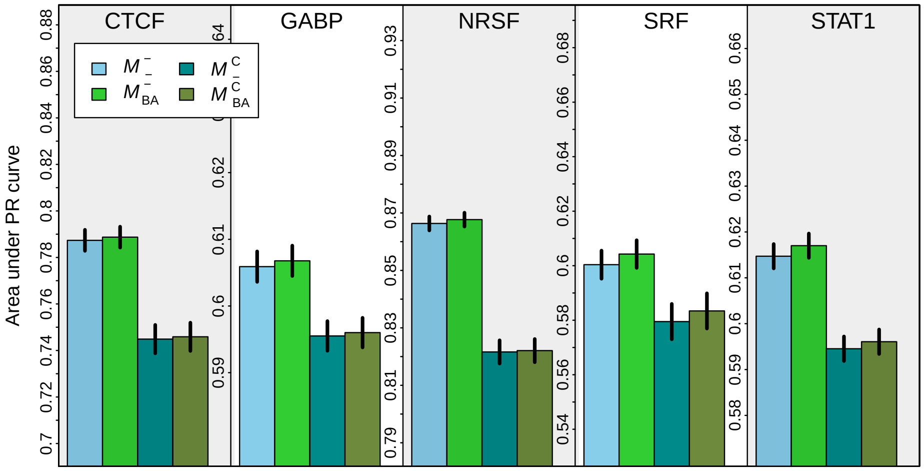

Figure S16: Classification performance measured by the area under the precision recall curve for pair-wise alignments of human and monkey. We compute the areas under the precision recall curves for each of the four classifiers consisting of foreground models $\mathcal{M}_{-}^{-}$, $\mathcal{M}_{\mathrm{BA}}^{-}$, $\mathcal{M}_{-}^{\mathrm{C}}$, and $\mathcal{M}_{\mathrm{BA}}^{\mathrm{C}}$ and background model $\mathcal{B}$ for each of the five data sets corresponding to the transcription factors CTCF, GABP, NRSF, SRF, and STAT1. We perform a stratified repeated random sub-sampling validation and show the means and their standard errors for each of the four models and each of the five data sets. The classification performance decreases in particular for the models with contamination bias ($\mathcal{M}_{-}^{\mathrm{C}}$ and $\mathcal{M}_{\mathrm{BA}}^{\mathrm{C}}$) compared to the results achieved for five species (see **Figure S2**). We find that $\mathcal{M}_{\mathrm{BA}}^{\mathrm{C}}$ yields a higher classification performance than $\mathcal{M}_{-}^{\mathrm{C}}$ and that $\mathcal{M}_{\mathrm{BA}}^{-}$ yields a higher classification performance than $\mathcal{M}_{-}^{-}$ for all five data sets which we find also in case of five species.

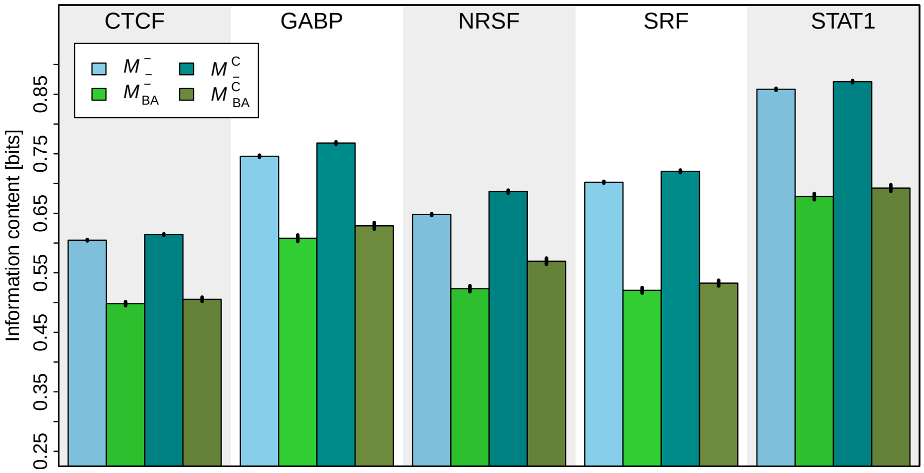

Figure S17: Information contents of the inferred motifs from data sets of pair-wise alignments of human and monkey. We compute the information contents for each of the inferred motifs from $\mathcal{M}_{-}^{-}$, $\mathcal{M}_{\mathrm{BA}}^{-}$, $\mathcal{M}_{-}^{\mathrm{C}}$, and $\mathcal{M}_{\mathrm{BA}}^{\mathrm{C}}$ for the data sets of each of the transcription factors CTCF, GABP, NRSF, SRF, and STAT1, each consisting of alignments of human and monkey only. We perform a stratified repeated random sub-sampling validation and show the means and their standard errors for each of the four models and each of the five data sets. The information contents estimated on data from human and monkey range from 0.5 to 0.95 and are higher compared to the results achieved for five species (0.25 to 0.65, see **Figure S3**). This indicates that the binding affinity bias is corrected to a lesser extend because the phylogenetic distance between human and monkey is not sufficient. However, as found in case of five species, the information contents of motifs inferred by $\mathcal{M}_{\mathrm{BA}}^{-}$ and $\mathcal{M}_{\mathrm{BA}}^{\mathrm{C}}$ are significantly smaller than the information contents of motifs inferred by $\mathcal{M}_{-}^{-}$ and $\mathcal{M}_{-}^{\mathrm{C}}$ in each of the five data sets, indicating an enrichment of high-affinity binding sites in all cases. In addition, the information contents of motifs inferred by $\mathcal{M}_{-}^{-}$ are higher than the information contents of motifs inferred by $\mathcal{M}_{\mathrm{BA}}^{\mathrm{C}}$ in all cases, indicating that the superposition of the contamination bias and the binding-affinity bias leads to a sharpening of the motifs.

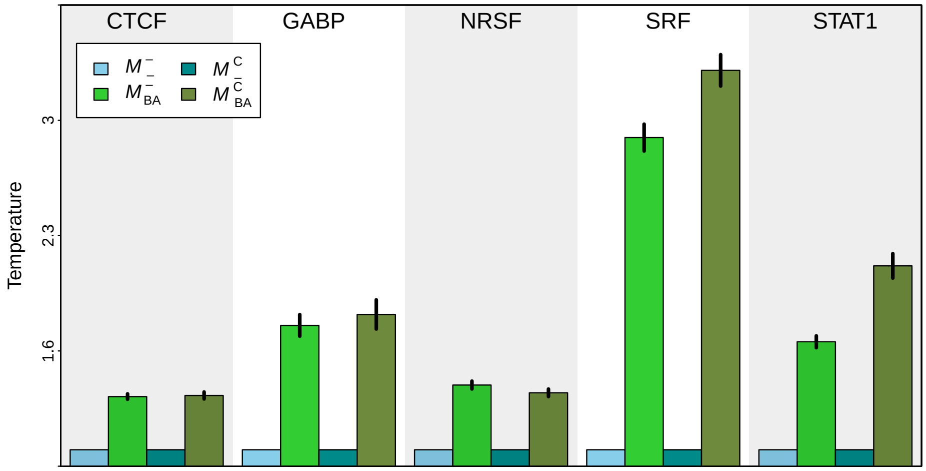

Figure S18: Inverse temperatures of $\mathcal{M}_{-}^{-}$,$\mathcal{M}_{-}^{\mathrm{C}}$,$\mathcal{M}_{\mathrm{BA}}^{-}$, and $\mathcal{M}_{\mathrm{BA}}^{\mathrm{C}}$ for pair-wise alignments of human and monkey. We plot the inverse temperatures β for each of the learned models $\mathcal{M}_{\mathrm{BA}}^{-}$ and $\mathcal{M}_{\mathrm{BA}}^{\mathrm{C}}$ for the data sets of each of the transcription factors CTCF, GABP, NRSF, SRF, and STAT1, each consisting of alignments of human and monkey only. We perform a stratified repeated random sub-sampling validation and show the means of β and their standard errors for the models $\mathcal{M}_{\mathrm{BA}}^{-}$ and $\mathcal{M}_{\mathrm{BA}}^{\mathrm{C}}$ for each of the five data sets together with the values of $\beta = 1$ for $\mathcal{M}_{-}^{-}$ and $\mathcal{M}_{-}^{\mathrm{C}}$. The average of the estimated inverse temperature range from $1.3 < \beta < 3.3$ and is typically higher compared to the results achieved for five species ($1.3 < \beta < 2.1$, see **Figure S4**). However, as found in case of five species, we find that β is significantly greater than 1 in both cases, indicating an enrichment of high-affinity binding sites in all five data sets.

4 Supplementary tables

		monkey	dog	cow	horse
**CTCF**	human	1.93e-17	3.96e-18	4.08e-18	3.96e-18
	monkey		4.33e-18	8.39e-18	3.96e-18
	dog			3.07e-12	4e-15
	cow				4.89e-18
**GABP**	human	1.83e-14	4.33e-18	3.96e-18	3.96e-18
	monkey		5.25e-17	3.96e-18	3.96e-18
	dog			1.07e-17	8.91e-18
	cow				0.445
**NRSF**	human	4.55e-16	3.96e-18	4.08e-18	3.96e-18
	monkey		3.96e-18	1.48e-17	3.96e-18
	dog			3.96e-18	3.37e-14
	cow				1.35e-17
**SRF**	human	1.88e-17	3.96e-18	3.96e-18	3.96e-18
	monkey		3.96e-18	3.96e-18	3.96e-18
	dog			4.46e-18	0.259
	cow				6.8e-18
**STAT1**	human	2.75e-17	3.96e-18	3.96e-18	3.96e-18
	monkey		3.96e-18	3.96e-18	3.96e-18
	dog			1e-17	3.63e-13
	cow				4.08e-18

Table S1: P-values for the differences of the information contents of the motifs in human, monkey, dog, cow, and horse calculated by the Wilcoxon Signed-Rank Test.

		$\mathcal{M}_{-}^{\mathrm{C}}$	$\mathcal{M}_{\mathrm{BA}}^{-}$	$\mathcal{M}_{\mathrm{BA}}^{\mathrm{C}}$
**CTCF**	$\mathcal{M}_{-}^{-}$	4.420e-16	5.342e-18	3.864e-18
	$\mathcal{M}_{-}^{\mathrm{C}}$		7.635e-18	4.410e-18
	$\mathcal{M}_{\mathrm{BA}}^{-}$			3.405e-05
**GABP**	$\mathcal{M}_{-}^{-}$	2.39e-12	6.87e-18	7.02e-18
	$\mathcal{M}_{-}^{\mathrm{C}}$		2.05e-17	7.29e-18
	$\mathcal{M}_{\mathrm{BA}}^{-}$			4.53e-11
**NRSF**	$\mathcal{M}_{-}^{-}$	1.97e-13	3.74e-18	7.37e-12
	$\mathcal{M}_{-}^{\mathrm{C}}$		5.67e-18	3.65e-18
	$\mathcal{M}_{\mathrm{BA}}^{-}$			4.45e-14
**SRF**	$\mathcal{M}_{-}^{-}$	0.899	1.79e-17	2.73e-11
	$\mathcal{M}_{-}^{\mathrm{C}}$		3.35e-13	2.26e-17
	$\mathcal{M}_{\mathrm{BA}}^{-}$			0.459
**STAT1**	$\mathcal{M}_{-}^{-}$	1.61e-17	3.63e-18	3.65e-18
	$\mathcal{M}_{-}^{\mathrm{C}}$		3.49e-18	3.54e-18
	$\mathcal{M}_{\mathrm{BA}}^{-}$			1.84e-13

Table S2: P-values for the differences of the areas under the receiver operating characteristics curves calculated by the Wilcoxon Signed-Rank Test.

		$\mathcal{M}_{-}^{\mathrm{C}}$	$\mathcal{M}_{\mathrm{BA}}^{-}$	$\mathcal{M}_{\mathrm{BA}}^{\mathrm{C}}$
**CTCF**	$\mathcal{M}_{-}^{-}$	6.11e-16	7.26e-17	1.35e-17
	$\mathcal{M}_{-}^{\mathrm{C}}$		4.48e-15	2.7e-16
	$\mathcal{M}_{\mathrm{BA}}^{-}$			8.07e-11
**GABP**	$\mathcal{M}_{-}^{-}$	2.78e-08	0.0175	4.45e-05
	$\mathcal{M}_{-}^{\mathrm{C}}$		0.877	0.025
	$\mathcal{M}_{\mathrm{BA}}^{-}$			1.8e-07
**NRSF**	$\mathcal{M}_{-}^{-}$	3.75e-13	3.81e-18	2.12e-16
	$\mathcal{M}_{-}^{\mathrm{C}}$		3.91e-18	3.78e-18
	$\mathcal{M}_{\mathrm{BA}}^{-}$			7.73e-15
**SRF**	$\mathcal{M}_{-}^{-}$	0.175	1.09e-17	3.87e-11
	$\mathcal{M}_{-}^{\mathrm{C}}$		3.4e-13	8.93e-18
	$\mathcal{M}_{\mathrm{BA}}^{-}$			0.224
**STAT1**	$\mathcal{M}_{-}^{-}$	4.67e-16	3.86e-18	3.84e-18
	$\mathcal{M}_{-}^{\mathrm{C}}$		3.78e-18	3.83e-18
	$\mathcal{M}_{\mathrm{BA}}^{-}$			2.01e-09

Table S3: P-values for the differences of the areas under the precision recall curves calculated by the Wilcoxon Signed-Rank Test.

		$\mathcal{M}_{-}^{\mathrm{C}}$	$\mathcal{M}_{\mathrm{BA}}^{-}$	$\mathcal{M}_{\mathrm{BA}}^{\mathrm{C}}$
**CTCF**	$\mathcal{M}_{-}^{-}$	3.96e-18	3.96e-18	3.96e-18
	$\mathcal{M}_{-}^{\mathrm{C}}$		3.96e-18	3.96e-18
	$\mathcal{M}_{\mathrm{BA}}^{-}$			3.96e-18
**GABP**	$\mathcal{M}_{-}^{-}$	5.04e-18	3.96e-18	3.96e-18
	$\mathcal{M}_{-}^{\mathrm{C}}$		3.96e-18	3.96e-18
	$\mathcal{M}_{\mathrm{BA}}^{-}$			2.3e-16
**NRSF**	$\mathcal{M}_{-}^{-}$	3.96e-18	3.96e-18	3.96e-18
	$\mathcal{M}_{-}^{\mathrm{C}}$		3.96e-18	3.96e-18
	$\mathcal{M}_{\mathrm{BA}}^{-}$			1.52e-17
**SRF**	$\mathcal{M}_{-}^{-}$	0.000739	3.96e-18	3.96e-18
	$\mathcal{M}_{-}^{\mathrm{C}}$		3.96e-18	3.96e-18
	$\mathcal{M}_{\mathrm{BA}}^{-}$			0.0602
**STAT1**	$\mathcal{M}_{-}^{-}$	4.89e-18	3.96e-18	3.96e-18
	$\mathcal{M}_{-}^{\mathrm{C}}$		3.96e-18	3.96e-18
	$\mathcal{M}_{\mathrm{BA}}^{-}$			1.18e-16

Table S4: P-values for the differences of the information contents calculated by the Wilcoxon Signed-Rank Test.

		$\mathcal{M}_{-}^{\mathrm{C}}$	$\mathcal{M}_{\mathrm{BA}}^{-}$	$\mathcal{M}_{\mathrm{BA}}^{\mathrm{C}}$
**CTCF**	$\mathcal{M}_{-}^{-}$		3.96e-18	3.95e-18
	$\mathcal{M}_{-}^{\mathrm{C}}$		3.96e-18	3.95e-18
	$\mathcal{M}_{\mathrm{BA}}^{-}$			2.89e-06
**GABP**	$\mathcal{M}_{-}^{-}$		3.96e-18	3.96e-18
	$\mathcal{M}_{-}^{\mathrm{C}}$		3.96e-18	3.96e-18
	$\mathcal{M}_{\mathrm{BA}}^{-}$			3.48e-17
**NRSF**	$\mathcal{M}_{-}^{-}$		3.95e-18	3.96e-18
	$\mathcal{M}_{-}^{\mathrm{C}}$		3.95e-18	3.96e-18
	$\mathcal{M}_{\mathrm{BA}}^{-}$			9.86e-05
**SRF**	$\mathcal{M}_{-}^{-}$		3.96e-18	3.96e-18
	$\mathcal{M}_{-}^{\mathrm{C}}$		3.96e-18	3.96e-18
	$\mathcal{M}_{\mathrm{BA}}^{-}$			3.03e-14
**STAT1**	$\mathcal{M}_{-}^{-}$		3.96e-18	3.96e-18
	$\mathcal{M}_{-}^{\mathrm{C}}$		3.96e-18	3.96e-18
	$\mathcal{M}_{\mathrm{BA}}^{-}$			3.96e-18

Table S5: P-values for the differences of the inverse temperatures calculated by the Wilcoxon Signed-Rank Test.

References

[1] Jospeh Felsenstein. Evolutionary trees from DNA sequences: a maximum likelihood approach. *Journal of Molecular Evolution*, 17(6):368–376, 1981.
